# A critical role for the chromatin remodeller CHD7 in anterior mesoderm during cardiovascular development

**DOI:** 10.1016/j.ydbio.2015.06.017

**Published:** 2015-09-01

**Authors:** Sophie Payne, Matthew J. Burney, Karen McCue, Nelo Popal, Sean M. Davidson, Robert H. Anderson, Peter J. Scambler

**Affiliations:** a Developmental Biology of Birth Defects Section, Institute of Child Health, University College London, 30 Guilford Street, London WC1N 1EH, UK; b The Hatter Cardiovascular Institute, University College London, 67 Chenies Mews, London WC1E 6HX, UK; c Institute of Genetic Medicine, Newcastle University, International Centre for Life, Central Parkway, Newcastle upon Tyne NE1 3BZ, UK

**Keywords:** Chromatin remodelling, Heart development, Congenital heart defects, CHARGE syndrome

## Abstract

CHARGE syndrome is caused by spontaneous loss-of-function mutations to the ATP-dependant chromatin remodeller chromodomain-helicase-DNA-binding protein 7 (CHD7). It is characterised by a distinct pattern of congenital anomalies, including cardiovascular malformations. Disruption to the neural crest lineage has previously been emphasised in the aetiology of this developmental disorder. We present evidence for an additional requirement for CHD7 activity in the *Mesp1*-expressing anterior mesoderm during heart development. Conditional ablation of *Chd7* in this lineage results in major structural cardiovascular defects akin to those seen in CHARGE patients, as well as a striking loss of cardiac innervation and embryonic lethality. Genome-wide transcriptional analysis identified aberrant expression of key components of the Class 3 Semaphorin and Slit–Robo signalling pathways in *Chd7*^*fl/fl*^*;Mesp1-Cre* mutant hearts. CHD7 localises at the *Sema3c* promoter *in vivo*, with alteration of the local chromatin structure seen following *Chd7* ablation, suggestive of direct transcriptional regulation. Furthermore, we uncover a novel role for CHD7 activity upstream of critical calcium handling genes, and demonstrate an associated functional defect in the ability of cardiomyocytes to undergo excitation–contraction coupling. This work therefore reveals the importance of CHD7 in the cardiogenic mesoderm for multiple processes during cardiovascular development.

## Highlights

• *Chd7* knockdown in the cardiogenic mesoderm leads to major structural heart defects.
• Cardiomyocyte excitation–contraction coupling is disrupted after Chd7 ablation.
• Components of Semaphorin, Slit–Robo and calcium handling pathways are dysregulated.
• CHD7 directly binds and alters the local chromatin structure at the *Sema3C* promoter.

## 1 Introduction

Heart development involves a series of tightly-regulated morphological changes, from the early establishment of a linear tube to looping, chamber formation, and septation (Buckingham et al., 2005). The importance of epigenetic regulation of gene expression programmes during this complex process is becoming increasingly apparent, with a range of chromatin-remodelling and histone-modifying factors now identified with roles in cardiovascular development and disease (Chang and Bruneau, 2012). Chromodomain-helicase-DNA-binding protein 7 (CHD7) is a large ATP-dependant nucleosome-remodelling protein. It co-localises with mono- and tri-methylated lysine 4 on histone H3 (Schnetz et al., 2009), and shows a high level of co-occupancy with p300 binding sites in embryonic stem cells, indicating it may regulate gene expression through enhancer-binding (Schnetz et al., 2010). Chromatin remodelling by CHD7 is involved in the transcriptional regulation of key developmental processes, including great vessel morphogenesis (Randall et al., 2009), neural crest cell (NCC) formation (Bajpai et al., 2010), and neuronal differentiation (Engelen et al., 2011; Layman et al., 2009).

Haploinsufficiency for *CHD7* causes human CHARGE syndrome in over 90% of clinical cases (Vissers et al., 2004). CHARGE is a developmental disorder characterised by a specific pattern of defects, including ocular coloboma, heart malformations, atresia of the choanae, growth retardation, genital hypoplasia and ear abnormalities (Hall, 1979). A wide range of congenital heart defects are seen in approximately 74–77% of CHARGE patients (Zentner et al., 2010; Corsten-Janssen et al., 2013), with an overrepresentation of atrioventricular septal defects (AVSDs) and outflow tract (OFT) defects (Corsten-Janssen et al., 2013). *CHD7* mutations have also been detected in a large-scale study of *de novo* mutations in human sporadic congenital heart defects (Zaidi et al., 2013).

Investigation of *Chd7*-null embryos indicates that CHD7 interacts with BMP-activated SMAD1/5/8 to regulate expression of cardiogenic BMP target genes such as *Nkx2.5, Gata4 and Tbx20* during early cardiogenesis and chamber formation (Liu et al., 2014). However, C*hd7*^*−/−*^ mouse mutants die at embryonic day (E)10.5 due to p53-dependant growth failure (Van Nostrand et al., 2014), limiting a full assessment of heart development. *Chd7*^*+/*^^−^ mice are viable and phenocopy a number of aspects of CHARGE syndrome, including partial penetrance of ventricular septal defects (VSDs) and interrupted aortic arch type B (IAA-B) (Randall et al., 2009; Bosman et al., 2005; Hurd et al., 2007), although the full spectrum and severity of CHARGE cardiovascular malformations are not seen. We have therefore utilised a conditional knockdown model to investigate further the role of CHD7 and its tissue-specific requirements during heart development.

There are three known developmental origins for the cells that make up the mature heart: the cardiogenic mesoderm, from which the myocardium and endocardium are derived (Saga et al., 1999); cardiac NCCs, which contribute to OFT septation and great vessel development (Creazzo et al., 1998; Waldo et al., 2005); and the proepicardial organ, which provides components of the coronary vasculature system (Merki et al., 2005). CHARGE syndrome is often classified as a disease arising from maldevelopment of NCCs, known as a neurocristopathy (Etchevers et al., 2006), and CHD7 activity has been shown to have an essential role in the activation of the NCC transcriptional circuitry (Bajpai et al., 2010). We show for the first time that loss of *Chd7* in the early cardiogenic mesoderm, driven by *Mesp1-Cre*, results in major structural defects and gene dysregulation, leading to cardiac failure and embryonic lethality around E15.5. Endocardial-specific ablation of *Chd7* also results in septation and great vessel defects, indicating disruption to endocardium development is contributing to these malformations. CHD7 action lies upstream of key extracellular signalling molecules, including components of the Semaphorin and Slit–Robo pathways, as well as cardiac calcium handling genes, with consequences for excitation–contraction coupling in cardiomyocytes. Together, these results indicate that disruption to CHD7 activity in the cardiogenic mesoderm significantly contributes to the cardiovascular malformations seen in CHARGE patients.

## 2 Results and discussion

### 2.1 Embryonic lethality and great vessel defects following mesodermal ablation of *Chd7*

**Fig. 1:**
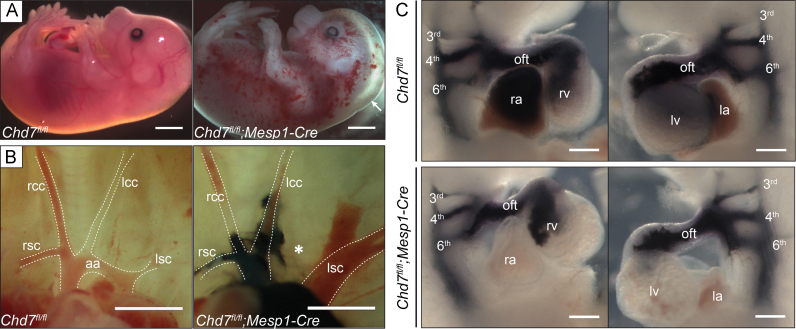
Major cardiovascular defects in *Chd7*^*fl/fl*^*;Mesp1-Cre* embryos. (A) At E15.5, *Chd7*^*fl/fl*^*;Mesp1-Cre* embryos showed severe oedema (arrow) and/or haemorrhaging compared to littermate *Chd7*^*fl/fl*^ controls. (B) Normal great vessel configuration was seen in *Chd7*^*fl/fl*^ embryos, whilst 21% of *Chd7*^*fl/fl*^*;Mesp1-Cre* embryos showed interrupted aortic arch type B (IAA-B) when examined at E15.5 (star). India ink injection was used on the *Chd7*^*fl/fl*^*;Mesp1-Cre* embryo shown here to better visualise the great vessels. (C) Ink injection at E10.5 was used to visualise the developing left and right 3rd, 4th and 6th pharyngeal arch arteries (PAAs) in *Chd7*^*fl/fl*^ (top panel) and *Chd7*^*fl/fl*^*;Mesp1-Cre* (bottom panel) embryos. All *Chd7*^*fl/fl*^*;Mesp1-Cre* embryos examined showed normal PAA development (*n*=14). *Scale bars represent 1* *mm (A), 0.5* *mm (B) or 0.2* *mm (C). rsc/lscindicates right/left subclavian artery; rcc/lcc, right/left common carotid artery; aa, aortic arch; oft, outflow tract; ra/la*, *right/left atrium; rv/lv, right/left ventricle.*

Mice homozygous for a conditional floxed *Chd7* allele (*Chd7*^*fl/fl*^) were crossed with *Chd7*^*fl/+*^*;Mesp1-Cre* mice, to homozygously ablate *Chd7* expression in the anterior mesoderm (Supplemental Fig. S1). *Mesp1-Cre* is expressed from E6.5 in the pharyngeal and cardiac mesoderm, including both the first and second heart fields of mesodermal cardiac progenitors (Saga et al., 1999), and *in situ* hybridisation (ISH) confirmed loss of *Chd7* mRNA throughout the heart in E11.5 *Chd7*^*fl/fl*^*;Mesp1-Cre* embryos (Supplemental Fig. S2). No liveborn *Chd7*^*fl/fl*^*;Mesp1-Cre* pups were produced from this cross, and at E18.5 the number of *Chd7*^*fl/fl*^*;Mesp1-Cre* embryos collected were significantly below their expected Mendelian ratios (Supplemental Table S1). Approximately three-quarters of *Chd7*^*fl/fl*^*;Mesp1-Cre* embryos survived to E15.5, although over 90% (*n*=22) showed severe oedema and/or haemorrhaging, indicative of cardiac failure (Fig. 1A). This conditional model therefore bypasses the p53-dependant developmental delay and early embryonic lethality seen with constitutive *Chd7*^*−/*^^−^ mutants, allowing a fuller assessment of heart development and investigation into the role of CHD7 in septation and great vessel remodelling.

Examination of the great arteries at E15.5 revealed that 21% (*n*=14) of *Chd7*^*fl/fl*^*;Mesp1-Cre* embryos had interrupted aortic arch type B (IAA-B, Fig. 1B), compared to just 4% of heterozygous gene-trapped *Chd7*^*xk/+*^ embryos (Randall et al., 2009). The great vessel defects in *Chd7*^*xk/+*^ mutants were attributed to earlier pharyngeal arch artery (PAA) malformations seen at E10.5, and *Chd7* expression in the pharyngeal surface ectoderm – but not the mesoderm – was shown to be required for this early PAA morphogenesis (Randall et al., 2009). However, the PAAs were formed normally in all E10.5 *Chd7*^*fl/fl*^*;Mesp1-Cre* embryos examined (*n*=14, Fig. 1C), presumably as the ectodermal expression of *Chd7* is unaffected in this genotype. Instead, the IAA-Bs seen at E15.5 were likely due to a later PAA remodelling defect, indicating that *Chd7* is required in mesodermal derivatives for remodelling of the OFT and PAAs to form the mature configuration of the aortic arch and great vessels.

### 2.2 Major septation defects affect both the arterial and venous poles

**Fig. 2:**
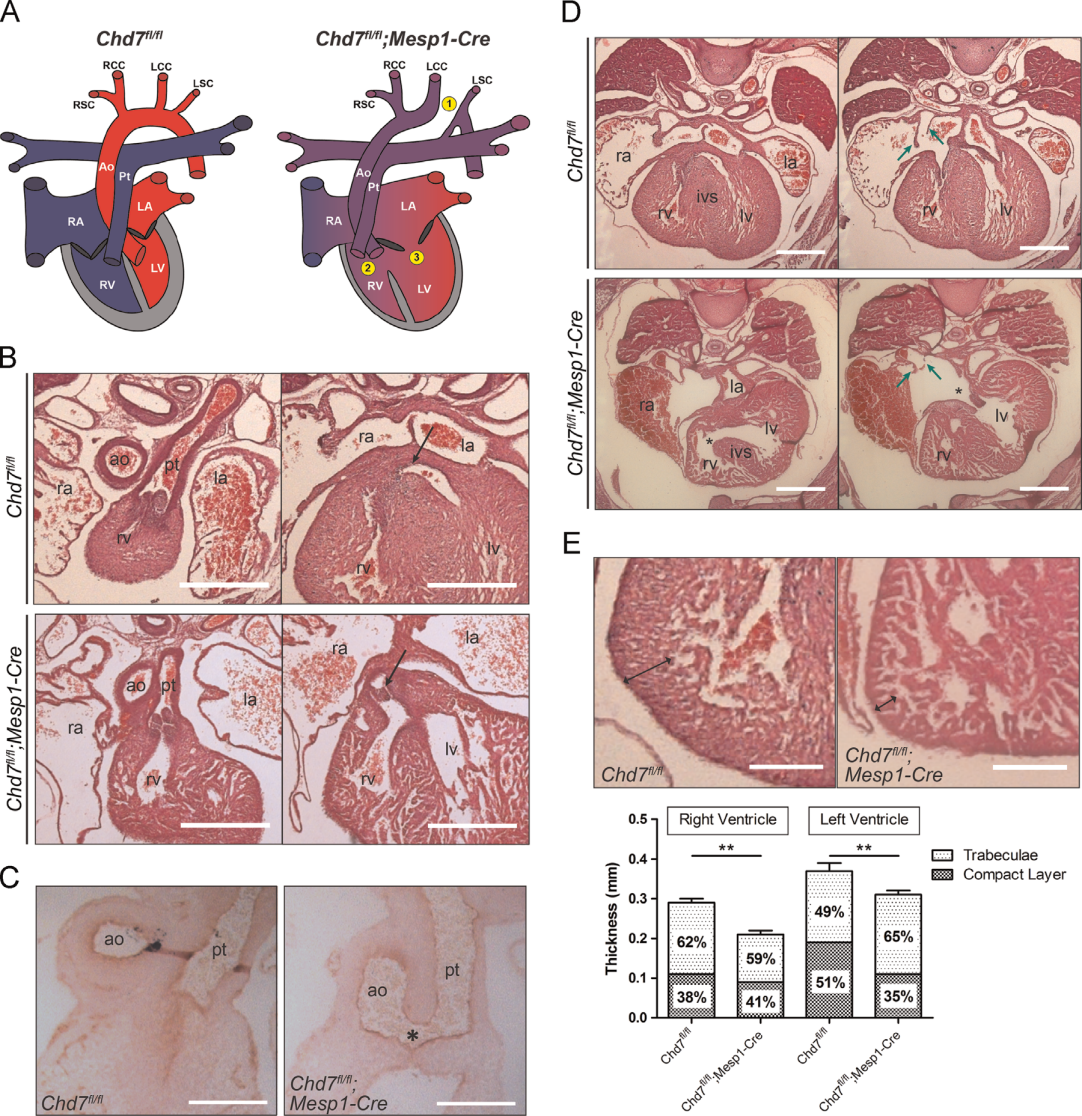
Structural defects affecting the arterial and venous poles. (A) Schematic to show the normal configuration of the heart in *Chd7*^*fl/fl*^ controls, with complete separation of the pulmonary (blue) and systemic (red) circulatory systems, compared to major septation and alignment defects seen in *Chd7*^*fl/fl*^*;Mesp1-Cre* hearts. These included interrupted aortic arch type B (IAA-B-1), double outlet right ventricle (DORV-2) and double inlet left ventricle (DILV-3). (B) Transverse H&E sections through the heart at E15.5 showed normal morphology in *Chd7*^*fl/fl*^ embryos, whilst DORV was present in 60% of *Chd7*^*fl/fl*^*;Mesp1-Cre* hearts, whereby both the pulmonary trunk (left panel) and the base of the aorta (black arrow, right panel) arise from the right ventricle. (C) Common arterial trunk (CAT), where the outflow tract is not fully septated into a separate aorta and pulmonary trunk, was seen in a *Chd7*^*fl/fl*^*;Mesp1-Cre* embryo collected at E13.5. An aortopulmonary window (star) can be seen, above a common set of valves. Normal OFT septation was seen in all *Chd7*^*fl/fl*^ controls. (D) All *Chd7*^*fl/fl*^*;Mesp1-Cre* hearts had DILV, including inter-ventricular communication (star, left panel), common AV valves (star, right panel) and poor formation of the venous valves (green arrows). (E) The compact myocardial layer of the ventricular wall was thin when compared to *Chd7*^*fl/fl*^ hearts (compare double-headed arrows). The bar graph shows the mean thickness of the compact and trabecular layers of the ventricles, indicating that the overall thickness of both the right and left ventricles was significantly reduced in *Chd7*^*fl/fl*^*;Mesp1-Cre* hearts. The percentage of the overall wall thickness that each layer comprised is also indicated on the graph, showing the compact layer is particularly affected in the left ventricle of the mutants. *^⁎⁎^ p<0.01 (calculated using unpaired student t test). Scale bars represent 0.5 mm. ao indicates aorta; pt, pulmonary trunk; ra/la, right/left atrium; rv/lv, right/left ventricle; ivs, inter-ventricular septum*.

Correct alignment and septation of the developing cardiac components ensures the complete separation of the pulmonary and systemic circulations, which is vital for effective cardiovascular function. Haematoxylin and eosin (H&E) staining of sections showed major structural defects in *Chd7*^*fl/fl*^*;Mesp1-Cre* hearts (summarised in Fig. 2A and Table 1). Sixty percent of hearts examined at E15.5 (*n*=10) had double outlet arising from the right ventricle (DORV, Fig. 2B), and common arterial trunk (CAT) was seen in one *Chd7*^*fl/fl*^*;Mesp1-Cre* embryo at E13.5 (*n*=10, Fig. 2C). Whilst these malformations are indicative of an abnormality in the addition of second heart field (SHF)-derived cells to the arterial pole, Islet-1 staining showed similar distributions of undifferentiated SHF progenitors in the developing OFTs of control *Chd7*^*fl/fl*^ and mutant *Chd7*^*fl/fl*^*;Mesp1-Cre* hearts (Supplemental Fig. S3). It may therefore be the subsequent differentiation of these SHF-derived cells in *Chd7*^*fl/fl*^*;Mesp1-Cre* hearts that is underlying the observed OFT defects.

All hearts examined at E15.5 also had double inlet left ventricle (DILV, Fig. 2D), a severe form of AVSD whereby both atria are connected through a common atrioventricular (AV) valve to a dominant left ventricle, with no access from the AV junction to the incomplete right ventricle. Related to this malformation, an inter-ventricular communication was observed in all hearts, and the venous valves were absent or poorly formed. Furthermore, the ventricular mural myocardium was poorly compacted in *Chd7*^*fl/fl*^*;Mesp1-Cre* hearts (Fig. 2E), although the development of the epicardium looked normal (Supplemental Fig. S4).

**Fig. 3:**
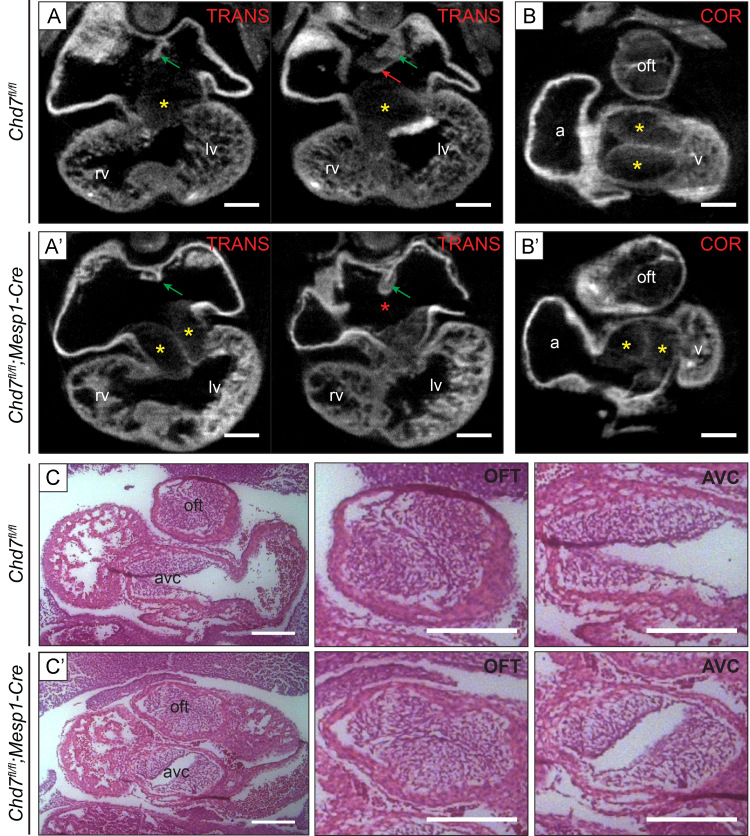
E11.5 *Chd7*^*fl/fl*^*;Mesp1-Cre* hearts show loss of the vestibular spine and misaligned endocardial cushions. (A) Transverse (TRANS) OPT sections through a *Chd7*^*fl/fl*^ heart showed formation of the primary atrial septum (green arrows) and the vestibular spine (red arrow). The endocardial superior and inferior AV cushions were seen in separate planes (yellow stars). In the *Chd7*^*fl/fl*^*;Mesp1-Cre* heart (A′) the atrial septum formed as expected (green arrows), but the vestibular spine was completely absent (red star). The arrangement of the endocardial cushions was also grossly abnormal, with both seen adjacently in the same plane (yellow stars). (B) Digital re-slicing was used to produce coronal (COR) sections of the same hearts, which highlighted the abnormal positioning of the endocardial cushions following mesodermal *Chd7* ablation (B′, yellow stars). (C) Morphology was also examined using H&E staining of coronal sections at E11.5, to better view the cushions of the OFT and AV canal. Despite their abnormal positioning within the AV canal, mesenchymal population of the cushions does not appear to be affected in mutants. *Scale bars represent0.2* *mm. oft indicates outflow tract; rv, right ventricle; lv, left ventricle; a, atrium; v, ventricle; avc, atrioventricular canal*.

The presence of AVSD and common AV valves is indicative of a failure of the vestibular spine (also known as the dorsal mesenchymal protrusion) to form at the venous pole of the heart. It is this spine that drives the separation of the AV canal into its left and right components and carries forward the inferior ends of the venous valves (Anderson et al., 2003). To investigate this further, optical projection tomography (OPT) was used to examine hearts at E11.5, which showed the vestibular spine was absent or reduced in size in *Chd7*^*fl/fl*^*;Mesp1-Cre* hearts (Fig. 3A). This imaging also showed grossly abnormal positioning of the endocardial cushions, which were markedly rotated within the AV canal compared to the control hearts (Fig. 3B). These local tissue swellings contribute to the septation of the chambers to form the functional four-chambered heart, and a hypocellular AV cushion defect has been reported in *Chd7*-null embryos at E9.5 and E10.5 (Liu et al., 2014). Despite their abnormal positioning, a similar hypocellular defect was not seen in the cushions of *Chd7*^*fl/fl*^*;Mesp1-Cre* hearts at E10.5 or E11.5 (Fig. 3C and Supplemental Fig. S5), indicating that mesenchymal population of the cushions is not affected in these conditional mutants.

The great vessel, OFT and septation defects we observed in *Chd7*^*fl/fl*^*;Mesp1-Cre* hearts correlate well with the reported clinical CHARGE malformations (Corsten-Janssen et al., 2013). However, whilst AVSD is commonly seen, DILV is not usually associated with CHARGE syndrome. This difference likely reflects dosage dependence for *Chd7* expression, with homozygous deletion of *Chd7* in the mesoderm resulting in a greater reduction in CHD7 activity than occurs in CHARGE patients. Similarly, lack of haploinsufficiency in our heterozygous mesodermal conditional mutants indicates that hemizygosity for the CHD7 protein is required in multiple tissue types for the CHARGE phenotype to become manifest. Interestingly, this combination of DILV with concordant ventriculo-arterial connections seen in the *Chd7*^*fl/fl*^*;Mesp1-Cre* hearts is analogous to the extremely rare condition in humans known as the “Holmes heart” (Dobell and Van Praagh, 1996).

### 2.3 Defective cardiac innervation and coronary vein development

**Fig. 4:**
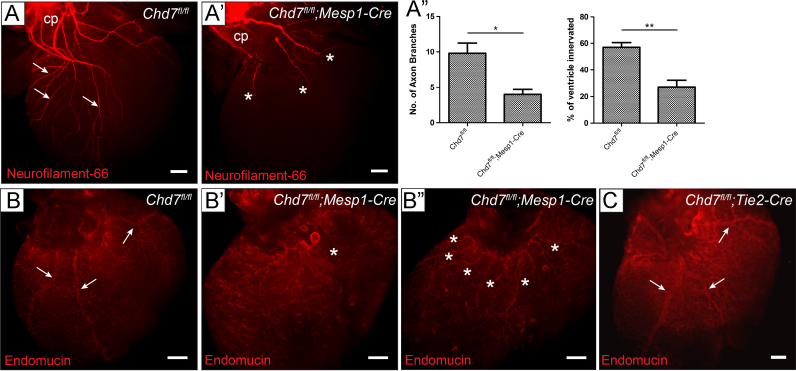
*Chd7*^*fl/fl*^*;Mesp1-Cre* hearts have defective innervation and coronary vein development. (A–A′) Wholemount immunostaining at E15.0 for Neurofilament-66 allowed visualisation of the sympathetic and parasympathetic innervation descending from the cardiac plexus and branching into the ventricles (white arrows). Severe truncation of axons was seen in *Chd7*^*fl/fl*^*;Mesp1-Cre* hearts (stars, A′). (A″) Quantification of the innervation defects: *Chd7*^*fl/fl*^*;Mesp1-Cre* hearts (*n*=4) showed significantly fewer axonal branch points viewed on the dorsal surface of the heart than in *Chd7*^*fl/fl*^ controls (*n*=5). The percentage of the dorsal ventricular surface area covered by the extending axons was also measured and found to be significantly reduced from a mean value of 57% in controls to 27% in mutants. (B–C) Dorsal view of E15.5 hearts immunostained with anti-Endomucin showed formation of three main coronary veins in control hearts (white arrows, B). In *Chd7*^*fl/fl*^*;Mesp1-Cre* hearts, patterning of the veins was clearly disrupted, with either severely truncated of vessels or additional smaller vessels seen (stars, B′ and B″). However, coronary vein development was normal in *Chd7*^*fl/fl*^*;Tie2-Cre* hearts (arrows, C). *^⁎^ p<0.05, ^⁎⁎^ p<0.01 (unpaired student t test). Scale bars represent2* *mm. Cp indicates cardiac plexus*.

To characterise further the cardiac phenotype following mesodermal *Chd7* ablation, the innervation and vascularisation of these hearts was investigated. Wholemount immunostaining for Neurofilament-66 showed major truncation of the neuronal axons innervating *Chd7*^*fl/fl*^*;Mesp1-Cre* hearts (Fig. 4A). Both sympathetic and parasympathetic axons can be seen on the dorsal surface of the heart by E15.0, although they are predominantly sympathetic (Nam et al., 2013). They are derived from NCCs, which migrate to the dorsal aorta, differentiate into neurons and extend axonal projections into the cardiac tissue (Hasan, 2013). As *Chd7* expression is not deleted in NCCs in *Chd7*^*fl/fl*^*;Mesp1-Cre* embryos, this is indicative of a non-cell autonomous effect of mesodermal *Chd7* knockdown on the NCCs or neurons that contribute to cardiac innervation.

Major disruption to the coronary veins on the dorsal surface of the heart was also seen at E15.5 in *Chd7*^*fl/fl*^*;Mesp1-Cre* hearts by anti-Endomucin immunostaining, with either severe truncation of vessels or ectopic formation of multiple small veins (Fig. 4B). However, the coronary veins formed normally when *Chd7* was ablated specifically in endothelial cells using *Tie2-Cre* (Fig. 4C). Therefore, CHD7 activity within the myocardium again appears to have a non-cell-autonomous effect on the migratory endothelial cells required for the development of the coronary veins, presumably through transcriptional effects on extracellular guidance signalling from the myocardium to the venous endothelial cells.

### 2.4 Dissecting the role of CHD7 in the endocardium

**Fig. 5:**
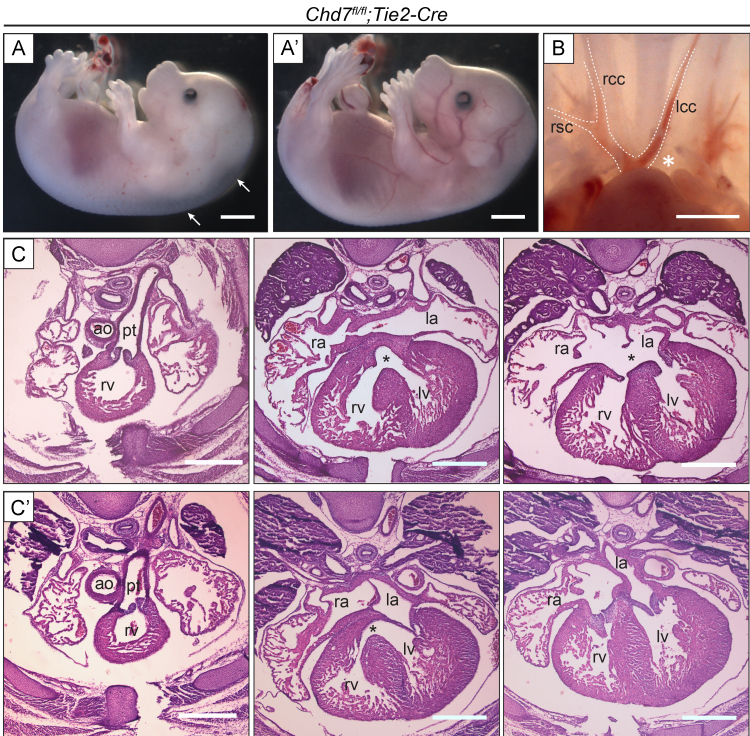
Endocardial ablation of *Chd7* results in less severe cardiovascular defects. (A) Mild oedema (left panel) was seen in just 18% of *Chd7*^*fl/fl*^*;Tie2-Cre* embryos collected at E15.5 (*n*=11), whilst the majority of these embryos had a normal external appearance (right panel). (B) One *Chd7*^*fl/fl*^*;Tie2-Cre* embryo examined showed interrupted aortic arch type B (IAA-B, star), as also seen in *Chd7*^*fl/fl*^*;Mesp1-Cre* embryos. (C–C′) Two examples of H&E transverse sections through E15.5 *Chd7*^*fl/fl*^*;Tie2-Cre* hearts, both of which show normal septation and alignment of the OFT (far left panels). The first (C) shows inter-ventricular communication associated with a balanced atrio-ventricular septal defect (AVSD, stars), and a thin myocardial wall in the right ventricle. The second (C′) shows a milder phenotype of ventricular septal defect (VSD, star in middle panel) with normal AV canal septation (right panel). *Scale bars represent 1 mm (A) or 0.5 mm (B–C). rsc indicates right subclavian artery; rcc/lcc, right/left common carotid artery; ao, aorta; pt, pulmonary trunk; rv/lv, right/left ventricle; ra/la, right/left atrium*.

The septation and trabeculation defects observed in *Chd7*^*fl/fl*^*;Mesp1-Cre* hearts could be due to abnormal development of the endocardium and its derivatives. We therefore addressed the importance of CHD7 activity in this lineage by utilising the pan-endothelial marker *Tie2-Cre* (Kisanuki et al., 2001) to drive endocardial-specific *Chd7* ablation. *Chd7*^*fl/fl*^*;Tie2-Cre* offspring were viable, and significantly fewer *Chd7*^*fl/fl*^*;Tie2-Cre* embryos collected at E15.5 showed oedema compared with *Chd7*^*fl;fl*^*;Mesp1Cre* embryos (see Table 2 and Fig. 5A). Furthermore, none showed haemorrhaging, indicating that CHD7 activity is not required cell autonomously for vessel integrity. Some great vessel and septation defects were present (Fig. 5B–C), and three out of eight *Chd7*^*fl/fl*^*;Tie2-Cre* embryos showed a similar myocardial non-compaction defect to *Chd7*^*fl;fl*^*;Mesp1-Cre* hearts. However, these malformations were seen at a lower penetrance and without the accompanying alignment defects seen following *Mesp1-Cre*-driven ablation. There were also no examples of DORV or CAT seen in *Chd7*^*fl/fl*^*;Tie2-Cre* embryos, suggesting that CHD7 activity in the endocardium is not involved in arterial pole septation.

Together, this data indicates that there is indeed a role for *Chd7* in the endocardium for AV canal septation and compaction of the myocardial walls. However, as the full *Chd7*^*fl/fl*^*;Mesp1-Cre* cardiac phenotype is not recapitulated in *Chd7*^*fl/fl*^*;Tie2-Cre* hearts, it may not be the only underlying mechanism. Reciprocal interactions between the endocardium and myocardium, which are both derived from *Mesp1*-expressing progenitors, are crucial during heart development (Tian and Morrisey, 2012). Therefore, it is likely the increased severity and frequency of defects seen in *Chd7*^*fl/fl*^*;Mesp1-Cre* hearts is due to a requirement for CHD7 activity in both lineages. Furthermore, as *Tie2-Cre* also marks the venous precursors of the lymphatic vasculature (Srinivasan et al., 2007), the absence of haemorrhage and the low penetrance of oedema in *Chd7*^*fl/fl*^*;Tie2-Cre* conditionals is not supportive of a major role for CHD7 in lymphatic development. Instead, poor cardiac function is most likely to be underlying these phenotypes in *Chd7*^*fl/fl*^*;Mesp1-Cre* embryos.

### 2.5 Differential gene expression following mesodermal *Chd7* ablation

To investigate the global transcriptional changes underlying the observed cardiovascular defects, microarrays were performed on mRNA extracted from dissected *Chd7*^*fl/fl*^*;Mesp1-Cre* versus *Chd7*^*+/+*^*;Mesp1-Cre* hearts. Gene expression was examined at both E11.5 and E13.5, when the growth of the vestibular spine, development of the endocardial cushions, and septation is normally occurring. Crucially, most cardiovascular defects occurred later in our conditional model than on the background of global *Chd7* knockdown and gross developmental delay, with no major loss of tissue or hypocellular phenotype in either E10.5 or E11.5 *Chd7*^*fl/fl*^*;Mesp1-Cre* hearts. Therefore, our analysis of a role for CHD7 subsequent to its previously-reported BMP-dependant role upstream of *Nkx2.5* should not be compromised by earlier defects (Liu et al., 2014). Furthermore, Western blot analysis of CHD7 protein in dissected wild-type hearts showed CHD7 was detectable until E13.5, whilst nuclear CHD7 protein was observed by immunohistochemistry throughout wild-type hearts at E10.5 and E11.5 (Supplemental Fig. S6). This indicates a later role for CHD7 in cardiovascular development than can be examined in constitutive *Chd7*^*−/−*^ mutants.

Full lists of genes with altered expression (log_2_ FC>0.5, *p*<0.05) in the heart at E11.5 or E13.5 following mesodermal deletion of *Chd7* are available in Supplemental Table S2. Multiple testing correction using the Benjamini–Hochberg procedure identified 31 genes that were significantly downregulated and 20 genes upregulated at E11.5, whilst 6 genes were downregulated and 3 upregulated at E13.5 (log_2_ FC>0.5, adj. *p*<0.05-see Table 3). This is consistent with previous reports that chromatin remodelling by CHD7 acts as a “transcriptional rheostat” to modulate the expression of its target genes in either a positive or negative direction (Schnetz et al., 2010). Gene ontology (GO) term clusters generated using the DAVID Bioinformatics resource (Huang et al., 2009) highlighted a number of processes relevant for later cardiac development and the defects observed in the *Chd7*^*fl/fl*^*;Mesp1-Cre* hearts (Supplemental Tables S3–6). Interestingly, many of the GO clusters involved extracellular signalling pathways, such as cell surface receptor linked signal transduction and G-protein coupled receptor signalling pathways, rather than direct transcriptional regulators such as *Nkx2.5* and *Tbx20*, which are downregulated in earlier *Chd7*-null hearts (Liu et al., 2014).

We chose to investigate further components of the Semaphorin and Slit–Robo extracellular signalling pathways that were disrupted in the microarray datasets, alongside genes involved in the excitation–contraction coupling of cardiomyocytes, due to their known roles in cardiovascular development and relevance to the observed defects in *Chd7*^*fl/fl*^*;Mesp1-Cre* hearts (see Supplemental Table S7).

### 2.6 Disruption to Class 3 Semaphorin and Slit–Robo signalling pathways

**Fig. 6:**
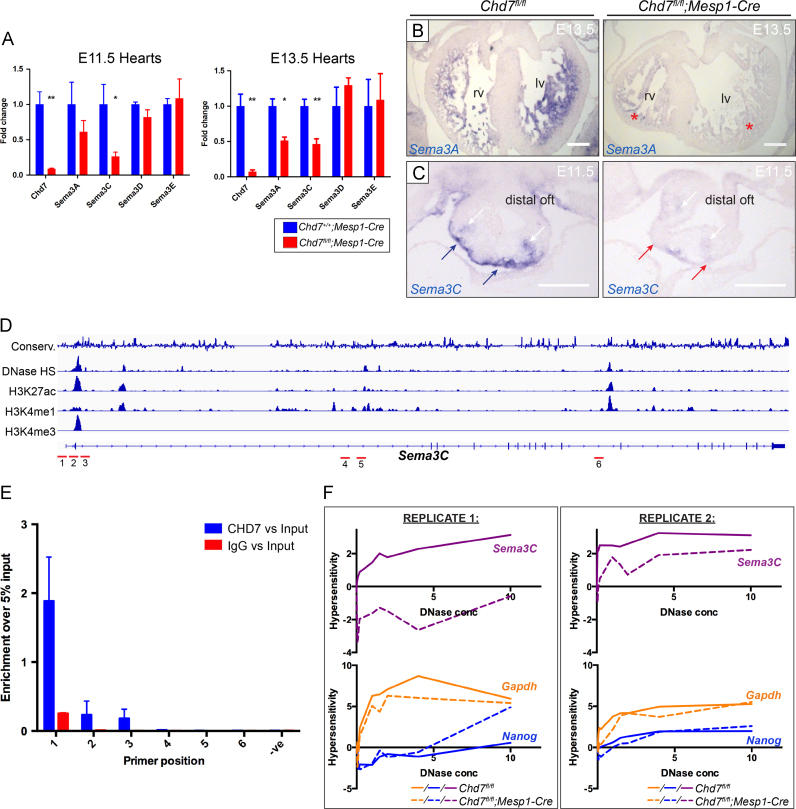
CHD7 regulates Class 3 Semaphorin expression in the developing heart. (A) Real-time PCR comparing expression of Class 3 Semaphorin genes in *Chd7*^*+/+*^*;Mesp1-Cre* and *Chd7*^*fl/fl*^*;Mesp1-Cre* dissected hearts at E11.5 and E13.5. Supplemental Table S7 shows the microarray results for these genes for comparison. (B) At E13.5, trabecular expression of *Sema3A* was seen by ISH on control *Chd7*^*fl/fl*^ sections, with significant reduction in *Chd7*^*fl/fl*^*;Mesp1-Cre* hearts (red stars). (C) ISH at E11.5 showed *Sema3C* expression in the myocardial cuff (blue arrows), which was strongly downregulated at the distal end of the OFT in *Chd7*^*fl/fl*^*;Mesp1-Cre* hearts (red arrows). *Sema3C* expression in the cardiac neural crest was also slightly reduced in mutants (white arrows). (D) ENCODE data around the *Sema3c* locus was used to design primer sets (denoted in red) to overlap with areas of DNase I hypersensitivity and histone modification marks associated with suspected promoter and enhancer regions. (E) ChIP-PCR demonstrated CHD7 binding around the promoter of *Sema3C* at E11.5 using Primer pair 1. (F) DNase I hypersensitivity assay showed greater chromatin compaction in *Chd7*^*fl/fl*^*;Mesp1-Cre* hearts (dotted line) than littermate controls (solid line) at the *Sema3c* promoter. Little change was seen at the *Gapdh* or *Nanog* promoters, although *Gapdh* displayed greater hypersensitivity, correlating with its constitutive expression. Individual replicates are shown, as it was not possible to normalise across separate experiments. ⁎ *p<0.05,* ⁎⁎ *p<0.01 (unpaired studentttest). Scale bars represent0.2* *mm. oft indicates outflow tract; rv, right ventricle; lv, left ventricle*.

The signalling glycoprotein Semaphorin 3A, encoded by *Sema3a*, acts as a potent neural chemorepellent and has a key role in sympathetic innervation patterning of the heart: homozygous null *Sema3a*^*−/*^^−^ mice show disruption to cardiac innervation leading to sinus bradycardia (Ieda et al., 2007). *Sema3a* expression was significantly downregulated in both the E11.5 and E13.5 microarray datasets (Table 3), and RT-PCR and ISH confirmed the trabecular expression of *Sema3a* seen at E13.5 was drastically reduced following mesodermal *Chd7* ablation (Fig. 6A and B). This downstream loss of *Sema3A* expression may be contributing to the innervation defects seen in *Chd7*^*fl/fl*^*;Mesp1-Cre* hearts. Alternatively, given the location of its expression at this stage, it may be influencing the trabeculation and compaction of the ventricles, which are also affected in *Chd7*^*fl/fl*^*;Mesp1-Cre* hearts.

Loss-of-function mutations of *SEMA3A* are associated with human Kallmann Syndrome (Hanchate et al., 2012), a disorder having defects overlapping with CHARGE. Of interest, non-synonymous *SEMA3A* variations have been found in 3 of 45 CHD7-negative CHARGE patients, and *Sema3a* expression is lost after morpholino-knockdown of *Chd7* in *Xenopus (*Schulz et al., 2014*)*. This work therefore strengthens the link between *CHD7* and *SEMA3A* and indicates it is relevant in the context of heart development and cardiac innervation. Furthermore, it is in agreement with, and extends, a recent report that *Sema3a* and *Sema3c* have diminished expression in whole E9.5 *Chd7*^*−/*^^−^ embryos (Schulz et al., 2014).

Downregulation of *Sema3c* expression was also seen in the microarrays and qRT-PCR validation. ISH at E11.5 confirmed *Sema3c* expression is specifically lost in the myocardial cuff at the distal end of the OFT in *Chd7*^*fl/fl*^*;Mesp1-Cre* hearts (Fig. 6C). Notably, *Sema3c*^*-/-*^ embryos have defects in the morphogenetic patterning of the aortic arch and OFT that significantly overlap with *Chd7*^*fl/fl*^*;Mesp1-Cre* hearts, including CAT, DORV and IAA-B (Feiner et al., 2001). Earlier PAA formation is also unaffected in these embryos, as we observe in *Chd7*^*fl/fl*^*;Mesp1-Cre* embryos. Chromatin immunoprecipitation (ChIP) for CHD7 followed by qRT-PCR performed on chromatin from dissected E11.5 hearts showed enrichment for CHD7 specifically at the promoter region of *Sema3c* (Fig. 6D and E). DNase I hypersensitivity assays, designed to test the accessibility of this region of DNA as a readout of chromatin remodelling activity, also showed a significant reduction in sensitivity to increasing concentrations of DNase I in *Chd7*^*fl/fl*^*;Mesp1-Cre* hearts (Fig. 6F). This is the first direct target for CHD7 regulation in the heart to be identified *in vivo*, and supports our proposal that diminished Semaphorin 3C signalling in the OFT contributes to the defects seen in *Chd7*^*fl/fl*^*;Mesp1-Cre* hearts.

**Fig. 7:**
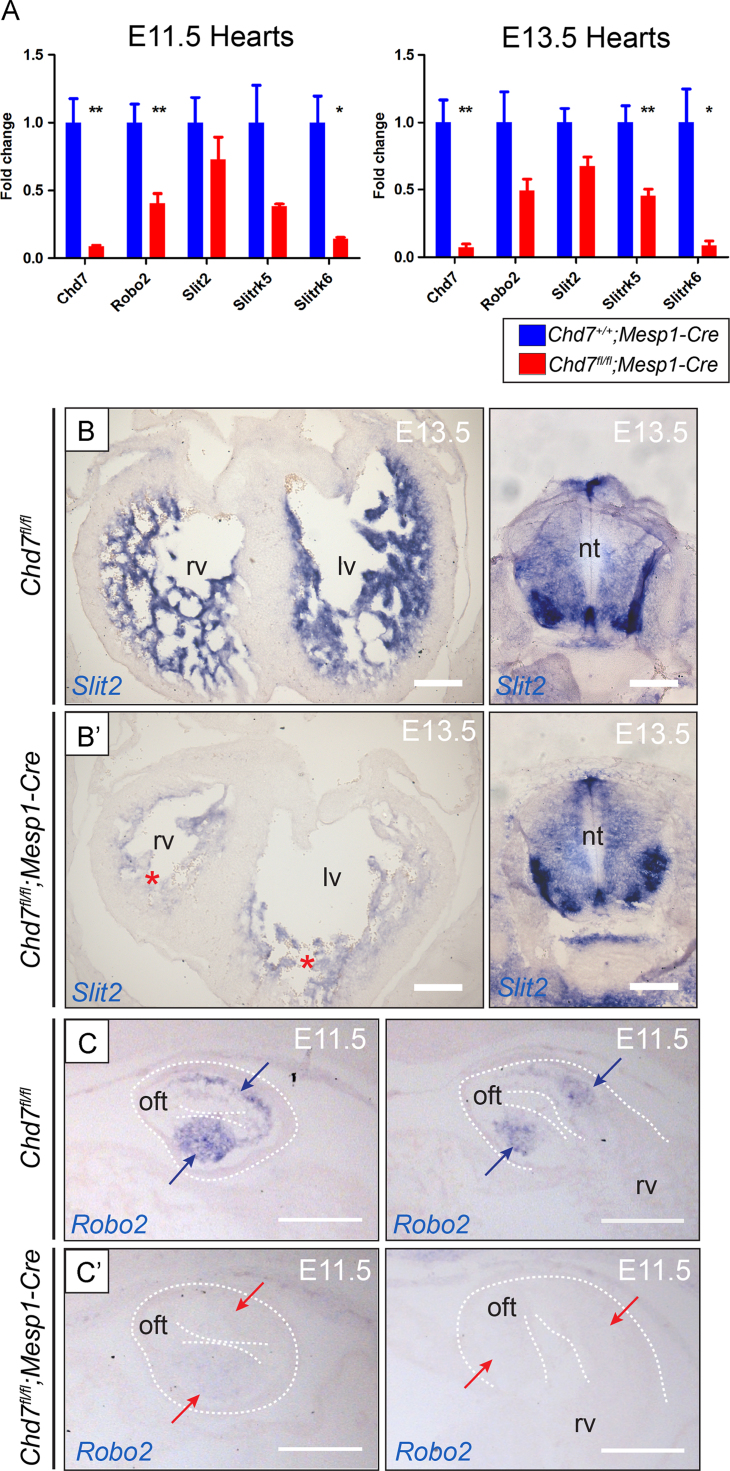
Disruption to the Slit–Robo pathway in *Chd7*^*fl/fl*^*;Mesp1-Cre* hearts. (A) Real-time PCR also showed disruption to Slit–Robo pathway genes in *Chd7*^*fl/fl*^*;Mesp1-Cre* hearts compared to *Chd7*^*+/+*^*;Mesp1-Cre* at E11.5 and E13.5. Supplemental Table S7 shows the microarray results for these genes. (B) *Slit2* expression is seen by ISH in the trabeculae at E13.5, with staining greatly reduced in *Chd7*^*fl/fl*^*;Mesp1-Cre* hearts (red stars). Sections through the neural tube of the same embryos are shown as an internal positive control for *Slit2* expression (right), showing the loss of expression is specific to *Mesp1*-derived tissue. (C) *Robo2* expression seen in the cushions of the proximal OFT (blue arrows) was lost in *Chd7*^*fl/fl*^*;Mesp1-Cre* hearts (red arrows). ^⁎^*p<0.05,*^⁎⁎^*p<0.01 (unpaired student t test).Scale bars represent0.2* *mm. oft indicates outflow tract; rv, right ventricle; lv, left ventricle; nt, neural tube*.

A number of components of the Slit–Robo signalling pathway were similarly downregulated following *Chd7* ablation, including *Slit2*, *Robo2*, *Slitrk5* and *Slitrk6* (Fig. 7A–C). The Slit and Robo protein families participate together in repulsive or attractive signalling to a range of migrating cells, including neurons (Rothberg et al., 1988), muscle precursors (Kramer et al., 2001), and endothelial cells (Zhang et al., 2009). The observation of truncated coronary veins after mesodermal, but not endothelial, deletion of *Chd7* therefore may be attributed, at least in part, to downregulation of Slit–Robo signalling from cardiomyocytes to migratory endothelial cells. Class 3 Semaphorin and Slit–Robo signalling have been shown to act synergistically for directional control of neuronal migration in the context of both ventral forebrain and corneal innervation (Hernandez-Miranda et al., 2011; Kubilus and Linsenmayer, 2010). The compound effect of disruption to components of both pathways could therefore impact on the innervation defects present in *Chd7*^*fl/fl*^*;Mesp1-Cre* hearts. Furthermore, the Slit–Robo pathway is crucial for ventricular septation, with VSDs observed at E14.5 in mice mutant for Slit2, Slit3, Robo1 and Robo1/Robo2 double mutants (Mommersteeg et al., 2015). Also of note, reduced outflow tract rotation was observed in the Robo1/Robo2 mutants (Mommersteeg et al., 2015).

Together, this data indicates CHD7 activity has a role upstream of the key Semaphorin and Slit–Robo extracellular signalling pathways in the heart around E11.5–E13.5, with CHD7 binding observed *in vivo* at the promoter of *Sema3c* suggesting direct regulation. Unfortunately, due to technical difficulties attempts at *in vivo* ChIP-seq or ChIP-PCR at other candidate loci were unsuccessful, so it remains to be determined whether other gene expression changes are the result of direct CHD7 activity.

### 2.7 Dysfunctional Ca^2+^ handling in *Chd7^fl/fl^;Mesp1-Cre* embryonic cardiomyocytes

**Fig. 8:**
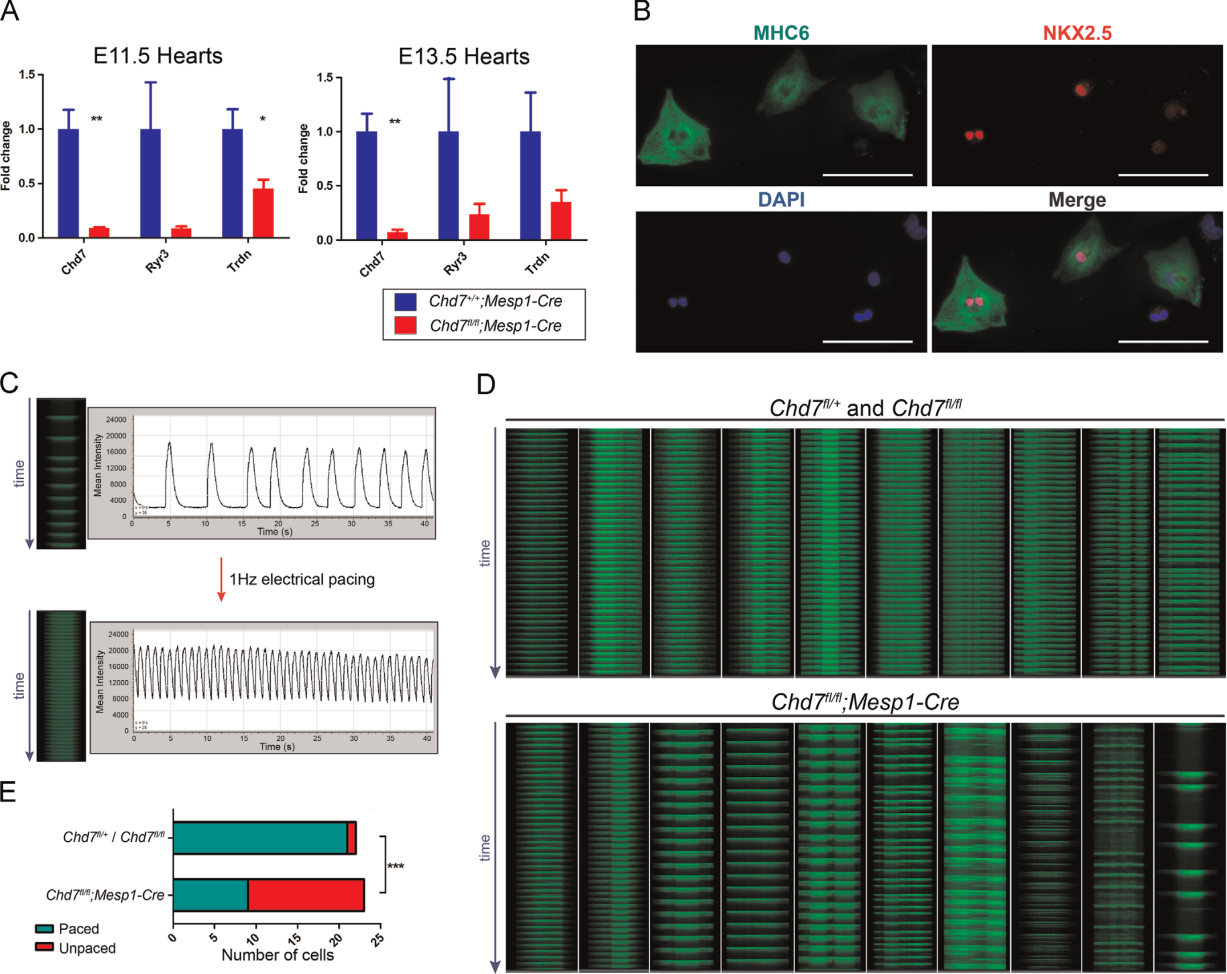
Impairment of excitation–contraction coupling in *Chd7*^*fl/fl*^*;Mesp1-Cre* cardiomyocytes. (A) Real-time PCR confirmed downregulation of selected Ca^2+^ handling genes in *Chd7*^*fl/fl*^*;Mesp1-Cre* dissected hearts at E11.5 and E13.5. Supplemental Table S7 shows the microarray results for these genes. (B) Immunocytochemistry showed cardiac-specific myosin heavy chain 6 (MHC6) and nuclear NKX2-5 levels in isolated E13.5 cardiomyocytes. (C) Rapid line scan analysis of Ca^2+^ transients over 40 s in individual cardiomyocytes showed irregular contractions, which could be paced using electrical field stimulation at 1 Hz. (D) Comparison of 10 representative control *Chd7*^*fl/+*^ or *Chd7*^*fl/fl*^ line scans (top panel) with *Chd7*^*fl/fl*^*;Mesp1-Cre* (bottom panel) showed a range of pacing defects in *Chd7*^*fl/fl*^*;Mesp1-Cre* cardiomyocytes. Only one control cell tested did not pace fully (far right of top panel). (E) Significantly fewer *Chd7*^*fl/fl*^*;Mesp1-Cre* cells responded to the electrical pacing compared to control cells. ^⁎^*p<0.05,*^⁎⁎^*p<0.01 (unpaired studentttest),*^⁎⁎⁎^*p<0.0001 (two-tailed Fisher's exact test).Scale bars in B represent0.1* *mm.*

In addition to the major morphological events involved in heart development, the ability of cardiomyocytes to undergo coordinated excitation–contraction coupling is essential for cardiovascular function. The ubiquitous messenger Ca^2+^ is the key signalling molecule for this process (Bers, 2002). Transcripts for the sarcoplasmic reticulum (SR) membrane-associated *Casq2, Trdn* and *Ryr3*, which play key roles in Ca^2+^-induced Ca^2+^ release during excitation–contraction coupling, were dysregulated, along with the L-type voltage-dependant Ca^2+^ channels *Cacng7, Cacna2d3* and *Cacna1e* (see Table S7 and Fig. 8A).

To determine whether the sum of these moderate transcriptional changes correlated with any functional effect, Ca^2+^ transients were visualised in embryonic cardiomyocytes *ex vivo*, using confocal microscopy for line scanning of individual cells over time (Fig. 8B and C). A striking difference was observed in the response to electrical field stimulation at 1 Hz between control (*Chd7*^*fl/fl*^ or *Chd7*^*fl/+*^) and *Chd7*^*fl/fl*^*;Mesp1-Cre* cardiomyocytes: 95% (*n*=22) of control cells responded to electrical pacing with regular Ca^2+^ transients recorded at the expected 1 s intervals, but only 39% (*n*=23) of *Chd7*^*fl/fl*^*;Mesp1-Cre* cells could be paced in this way (*p*<0.0001, Fig. 7D,E). This indicates the excitation–contraction coupling mechanism in cardiomyocytes is defective following ablation of *Chd7* in the cardiogenic mesoderm.

The loss of coordinated excitation–contraction coupling would be a major contributing cause of cardiac failure, which is likely underlying the severe oedema and embryonic lethality seen in *Chd7*^*fl/fl*^*;Mesp1-Cre* embryos. The importance of this cardiac failure is emphasised given the low level of oedema seen in *Chd7*^*fl/fl*^*;Tie2-Cre* embryos, as previously discussed. Ca^2+^ signalling is also an important factor in regulating cardiac morphogenesis, for example through secretion of cardiogenic signalling molecules and force generation (Puceat and Jaconi, 2005). To our knowledge, dysregulation of Ca^2+^ handling genes has not been remarked upon in any other transcriptomics analysis of hearts mutant for chromatin regulators. Interestingly, however, embryos mutant for the splicing regulator SRp38 also show disruption to *Trdn* and *Calq2* transcripts, leading to abnormal Ca^2+^ release from the SR and structural cardiac defects markedly similar to those seen in *Chd7*^*fl/fl*^*;Mesp1-Cre* hearts (Feng et al., 2009). Transcriptional changes to these genes and the resulting disruption to Ca^2+^ signalling therefore also likely contribute to the structural malformations observed in *Chd7*^*fl/fl*^*;Mesp1-Cre* hearts.

## 3 Conclusions

We have demonstrated a crucial role for CHD7 activity in the mesodermal lineage for the alignment, septation and maturation of the heart, as well as cardiac innervation, vascularisation, and remodelling of the great vessels. Ablation of *Chd7* in the cardiogenic mesoderm is sufficient to produce major structural defects and disrupted cardiomyocyte function, with the bypass of early lethality allowing the exploration of global transcriptional effects on later heart development. This analysis indicated a switch in the types of genes CHD7 regulates (directly or indirectly) as cardiogenesis progresses, from transcription factors such as NKX2-5, to extracellular signalling and Ca^2+^ handling genes. Furthermore, *Sema3c* is identified as a novel direct target of CHD7 in the developing heart *in vivo*. Therefore, disruption to transcriptional networks in both the cardiac neural crest and cardiogenic mesoderm likely contribute to the cardiovascular defects seen in CHARGE patients due to haploinsufficient loss of CHD7 activity.

## 4 Materials and methods

Further details of Materials and methods are included in the Supplemental material.

### 4.1 Mouse lines

Animal maintenance, husbandry and procedures were carried out in accordance with British Home Office regulations. The mouse lines used were: conditional *Chd7*^*fl*^ (MGI: 4433295, *Chd7*^*tm2a(EUCOMM)Wtsi*^), *Mesp1-Cre* (MGI: 2176467, *Mesp1*^*tm2(cre)Ysa*^), and *Tie2-Cre* (MGI: 2450311,*Tg(Tek-cre)1Ywa*). All lines were maintained on a C57Bl/6 J background. CD1 mice were used for CHD7 immunofluorescence and ChIP experiments. The date of observation of a vaginal plug was considered embryonic day E0.5.

### 4.2 Optical projection tomography

Embryos were fixed overnight in 4% PFA/PBS and mounted in low-melting agarose (Life Technologies). Samples were then trimmed and washed in 100% methanol followed by clearing in 2BA:1BB (2 parts benzyl alcohol to 1 part benzyl benzoate), before analysis using a Bioptonics OPT Scanner 3001M (MRC Technology, Edinburgh, UK). NRecon software (Skyscan NV) was used for image reconstruction from projections using a back-projection algorithm, and ImageJ was used for image analysis.

### 4.3 Microarrays

E11.5 or 13.5 hearts were dissected into RNase-free PBS and stored in Buffer RLT (Qiagen) at −80 °C until genotyped. Total RNA was isolated using RNeasy Mini kits (Qiagen), including the optional on-column DNase digestion step. Sense-strand cDNA was generated using the Ambion® WT Expression Kit and processed for hybridisation onto the Affymetrix Mouse Gene 1.0 ST Array according to manufacturer's protocols. Scanning was performed on the Affymetrix GeneChip Scanner. For each time point four biological replicates were run in each group: cDNA from four individual *Chd7*^*fl/fl*^*;Mesp1-Cre* hearts was compared to cDNA from four *Chd7*^*+/+*^*;Mesp1-Cre* hearts. The data was normalised with the RMA normalisation algorithm using the Affy R package (Gautier et al., 2004) provided as part of the Bioconductor libraries (Gentleman et al., 2004) and analysed for differential expression using LIMMA (Smyth, 2004). Data has been submitted to GEO (accession number GSE59963). The 100 significantly-altered genes with the highest fold changes from each up- or down-regulated list from the microarrays were uploaded to the DAVID Bioinformatics resource for GO analysis (Huang et al., 2009).

### 4.4 In situ hybridisation on paraffin sections

Plasmids for synthesising antisense RNA probes were kindly donated from the following sources: *Sema3a* and *Sema3c* from Christiana Ruhrberg; *Robo2* from Gail Martin; and *Slit2* from Bill Andrews (original clone from David Ornitz's lab). The *Chd7* probe was generated using TOPO® TA Cloning® Kits (Life Technologies) with previously reported primers (Bosman et al., 2005). Details for digoxigenin-labelling of probes, hybridisation and visualisation are included in the Supplemental material.

### 4.5 ChIP-PCR

Hearts were dissected from wildtype E11.5 CD1 embryos and sequentially fixed in 2 mM di(N-succinimidyl) glutarate (Sigma) for 45 min and 1% formaldehyde for 15 min. Hearts were quenched in 0.125 M glycine (Sigma) and washed in cold PBS. Tissue was lysed and passed through 21G and 23G needles five times each. Cells were pelleted by centrifugation and chromatin was released in 1% SDS buffer and sheared using a BioRuptor Sonicator (Diagenode), to an average size of 500 bp. All steps included 1× protease inhibitor cocktail (Roche).

ChIP was performed as described previously (Burney et al., 2013) with antibodies specific to CHD7 (#6505, Cell Signalling Technology) or non-specific rabbit IgG (Santa Cruz). Enrichment was analysed by real-time PCR performed using locus-specific primers (see Supplemental Material for sequences). Data were normalised to 5% input and compared to enrichment at non-specific loci.

### 4.6 DNase I hypersensitivity assay

Individual E11.5 hearts were dissected in cold PBS and incubated in 0.05% Trypsin-EDTA (Life Technologies) containing 10 µg/ml DNase I (Sigma) at 37 °C for 45 min. Trypsin was inhibited with 10% FBS (Life Technologies) and samples passed through a 23G needle three times. Cells were pelleted by centrifugation and nuclei released in lysis buffer (10 mM Tris pH7.4, 10 mM NaCl, 3 mM MgCl_2_, 0.1% NP40). Nuclei were treated with increasing concentrations of DNase I (Promega) at 37 °C for 10 min. 25 mM EDTA was added to stop reactions and samples were RNaseA and Proteinase K treated, before purification using a Qiaquick PCR purification kit. Eluted DNA was quantified using a Qubit fluorometer (Life Technologies) and diluted to the same concentration, before RT-PCR analysis using primers designed around gene promoters (see Supplemental material for sequences).

### 4.7 Measurement of Ca^2+^ transients in embryonic cardiomyocytes

Individual *Chd7*^*fl/fl*^, *Chd7*^*fl/+*^ or *Chd7*^*fl/fl*^*;Mesp1-Cre* E13.5 hearts were dissected in cold PBS and incubated in 0.05% Trypsin-EDTA (Life Technologies) containing 10 µg/ml DNase I (Sigma) at 37 °C for 45 min. Trypsin was inhibited with 10% FBS (Life Technologies) and samples passed through a 23G needle three times. Cells were pelleted by centrifugation and resuspended in 1 ml culture medium (DMEM, 10% FCS, 1× PenStrep, 1× Non-essential amino acids) before plating for 1.5 h to allow adherence of fibroblasts. The supernatant containing cardiomyocytes was then plated onto laminin-coated (Sigma) coverslip dishes, and cells cultured at 37 °C, 5% CO_2_ for 36 h. Plating efficiency and survival of *Chd7*^*fl/fl*^*;Mesp1-Cre* cells did not appear compromised.

For imaging, cells were washed once in imaging buffer (156 mM NaCl, 3 mM KCl, 2 mM MgSO_4_·7H_2_0, 1.25 mM K_2_HPO_4_, 2 mM CaCl_2_, 10 mM HEPES, 10 mM d-Glucose, pH7.4) before incubation at 37 °C for 10 min in imaging buffer/Opti-MEM® (50/50, Life Technologies) containing the Ca^2+^-sensitive fluorogenic dye 5 µM Cal-520™ (Stratech). A Leica SP5 confocal inverted microscope performed rapid line scan analysis (400 Hz) of Ca^2+^ transients in individual cells (ex: 488 nm, em: 500–600 nm). Cells producing Ca^2+^ transients were chosen randomly and were electrically paced by field stimulation at 1 Hz using platinum electrodes. Data was collected and analysed by averaging values across the line, over time, using Leica LAS AF software. “Paced” cells were classified as those in which Ca^2+^ transients were observed every 1 Hz over a 40 s interval, whereas in “non-paced” cells, Ca^2+^ transients were absent or unsynchronised. *p*-values were calculated using two-tailed Fisher's exact test.

## Author Contributions

SP, KM and PJS designed experiments, SP performed experiments and prepared the manuscript. MJB designed and performed ChIP and DNase assays, and analysed the microarray data. NP performed experiments and provided technical support. SD helped establish Ca^2+^ assays and RHA provided assistance with anatomical analysis of mutant hearts.

## Conflict of interest

The authors declare that they have no conflict of interest.

## Figures and Tables

**Table 1:** Cardiovascular defects observed at E15.5 in embryos from the *Chd7*^*fl/fl*^ x *Chd7*^*fl/+*^*;Mesp1-Cre* cross.

**Genotype**	**IAA-B**	**AVSD**	**DILV**	**DORV**	**Myocardial non-compaction**	**Venous valve defect**
***Chd7***^***fl/+***^	0/11	0/7	0/7	0/7	0/7	0/7
***Chd7***^***fl/fl***^	1/16	0/5	0/5	0/5	0/5	0/5
***Chd7***^***fl/+***^***;Mesp1-Cre***	1/9	0/4	0/4	0/4	0/4	0/4
***Chd7***^***fl/fl***^***;Mesp1-Cre***	3/14	10/10	10/10	6/10	8/10	9/10

IAA-B indicates interrupted aortic arch type B; AVSD, atrioventricular septal defect; DILV, double inlet left ventricle; DORV, double outlet right ventricle.

**Table 2:** Comparison of cardiovascular defects observed at E15.5 in *Chd7*^*fl/fl*^*;Tie2-Cre* and *Chd7*^*fl/fl*^*;Mesp1-Cre* embryos.

	***Chd7***^***fl/fl***^***;Tie2-Cre***	***Chd7***^***fl/fl***^***;Mesp1-Cre***
Oedema	18%	68%
Haemorrhage	0%	64%
IAA-B	12.5%	21%
AVSD	12.5%	100%
DILV	0%	100%
VSD only	25%	N/A
ASD only	12.5%	N/A
DORV	0%	60%
Myocardial non-compaction	37.5%	80%
Venous valve defects	0%	90%

Viable?	Yes – 5/8	No – 0/9

Genotypes were recorded as viable if live pups were recorded at P10, the numbers indicate the number of observed pups compared to the expected number based on Mendelian ratios. Numbers of *Chd7*^*fl/fl*^*;Mesp1-Cre* embryos examined are shown in Table 1. For *Chd7*^*fl/fl*^*;Tie2-Cre*, *n*=11 for external phenotypes and *n*=8 for great vessel and cardiac defects. IAA-B indicates interrupted aortic arch type-B; AVSD, atrioventricular septal defect; VSD, ventricular septal defect; ASD, atrial septal defect; DILV, double inlet left ventricle; DORV, double outlet right ventricle.

**Table 3:** Genes identified through microarray analysis with differential expression in *Chd7*^*fl/fl*^*;Mesp1-Cre* hearts following multiple testing correction (log_2_ FC>0.5, adj.*p.*value <0.05).

**Ensembl ID**	**Symbol**	**Chrom**	**Description**	**log**_**2**_** FC**	***p*-Value**	**Adj.*p*.value**
**E11.5**						
ENSMUSG00000041235	*Chd7*	4	Chromodomain helicase DNA binding protein 7	−1.68	6.11E−09	0.000212
ENSMUSG00000031558	*Slit2*	5	Slit homologue 2 (*Drosophila*)	−0.987	2.44E−08	0.000283
ENSMUSG00000035551	*Igfbpl1*	4	Insulin-like growth factor binding protein-like	1–1.30	1.08E−07	0.000939
ENSMUSG00000019787	*Trdn*	10	Triadin	−1.08	6.84E−07	0.00238
ENSMUSG00000039579	*Grin3 a*	4	Glutamate receptor ionotropic, NMDA3A	−0.972	6.21E−07	0.00238
ENSMUSG00000028883	*Sema3a*	5	Semaphorin 3A	−0.894	9.87E−07	0.00264
ENSMUSG00000018893	*Mb*	15	Myoglobin	−1.20	3.24E−06	0.00593
ENSMUSG00000024868	*Dkk1*	19	Dickkopf homologue 1 (*Xenopus laevis*)	−1.23	1.13E−05	0.0127
ENSMUSG00000004151	*Etv1*	12	ets Variant gene	1–0.614	1.11E−05	0.0127
ENSMUSG00000019906	*Lin7a*	10	Lin-7 homologue A (*C. elegans*)	−1.57	1.80E−05	0.0179
ENSMUSG00000044067	*Gpr22*	12	G protein-coupled receptor 22	−0.839	2.11E−05	0.0188
ENSMUSG00000052516	*Robo2*	16	Roundabout homologue 2 (*Drosophila*)	−0.907	2.38E−05	0.0201
ENSMUSG00000074491	*Clec4g*	8	C-type lectin domain family 4, member g	−0.986	2.52E−05	0.0203
ENSMUSG00000069171	*Nr2f1*	13	Nuclear receptor subfamily 2, group F, member	1–1.24	4.09E−05	0.0277
ENSMUSG00000016494	*Cd34*	1	CD34 antigen	−0.647	4.14E−05	0.0277
ENSMUSG00000073764	*Gm12888*	4	Predicted gene 12888	−0.837	4.38E−05	0.0282
ENSMUSG00000027463	*Slc52a3*	2	Solute carrier protein family 52, member	3–1.37	4.54E−05	0.0287
ENSMUSG00000007653	*Gabrb2*	11	Gamma-aminobutyric acid (GABA) A receptor, subunit beta	2–0.821	5.02E−05	0.0292
ENSMUSG00000020061	*Mybpc1*	10	Myosin binding protein C, slow-type	−1.25	5.32E−05	0.0303
ENSMUSG00000025488	*Cox8b*	7	Cytochrome c oxidase, subunit VIIIb	−1.17	6.16E−05	0.0323
ENSMUSG00000020682	*Mmp28*	11	Matrix metallopeptidase 28 (epilysin)	−0.564	6.10E−05	0.0323
ENSMUSG00000026678	*Rgs5*	1	Regulator of G-protein signalling	5–2.04	6.76E−05	0.0340
ENSMUSG00000049537	*Tecrl*	5	Trans-2,3-enoyl-CoA reductase-like	−0.643	6.93E−05	0.0341
ENSMUSG00000022519	*Srl*	16	Sarcalumenin	−0.550	7.58E−05	0.0346
ENSMUSG00000033737	*Fndc3c1*	*X*	Fibronectin type III domain containing 3C1	−1.07	8.05E−05	0.0354
ENSMUSG00000059742	*Kcnh7*	2	Potassium voltage-gated channel, subfamily H (eag-related), member 7	−0.992	8.03E−05	0.0354
ENSMUSG00000039057	*Myo16*	8	Myosin XVI	−0.717	0.000113	0.0446
ENSMUSG00000055639	*Dach1*	14	Dachshund 1 (*Drosophila*)	−0.673	0.000117	0.0450
ENSMUSG00000019851	*Perp*	10	PERP, TP53 apoptosis effector	−0.650	0.000118	0.0450
ENSMUSG00000061080	*Lsamp*	16	Limbic system-associated membrane protein	−0.577	0.000116	0.0450
ENSMUSG00000023328	*Ache*	5	Acetylcholinesterase	−0.799	0.000137	0.0497
ENSMUSG00000070867	*Trabd2b*	4	Predicted gene 12824	0.860	2.19E−06	0.00475
ENSMUSG00000021219	*Rgs6*	12	Regulator of G-protein signalling 6	0.895	4.07E−06	0.00615
ENSMUSG00000029322	*Plac8*	5	Placenta-specific 8	1.82	5.56E−06	0.00773
ENSMUSG00000025196	*Cpn1*	19	Carboxypeptidase N, polypeptide 1	1.47	6.39E−06	0.00854
ENSMUSG00000022103	*Gfra2*	14	Glial cell line derived neurotrophic factor family receptor alpha 2	1.19	8.69E−06	0.0108
ENSMUSG00000096555	*Olfr944*	9	Olfactory receptor 944	0.650	3.25E−05	0.0236
ENSMUSG00000069583	*Krtap12-1*	10	Keratin associated protein 12-1	0.558	4.05E−05	0.0277
ENSMUSG00000053519	*Kcnip1*	11	Kv channel-interacting protein 1	0.943	4.62E−05	0.0287
ENSMUSG00000051251	*Nhlh1*	1	Nescient helix loop helix 1	0.676	5.73E−05	0.0321
ENSMUSG00000095241	*Gm5478*	15		0.732	5.85E−05	0.0322
ENSMUSG00000097451	*Rian*	NA	NA	0.746	8.35E−05	0.0363
ENSMUSG00000027942	*4933434E20Rik*	3	RIKEN cDNA 4933434E20 gene	1.73	9.34E−05	0.0396
ENSMUSG00000031825	*Crispld2*	8	Cysteine-rich secretory protein LCCL domain containing 2	0.587	9.84E−05	0.0412
ENSMUSG00000029309	*Sparcl1*	5	SPARC-like 1	0.723	0.000106	0.0433
ENSMUSG00000070933	*Speer4d*	5	Spermatogenesis associated glutamate (E)-rich protein 4d	0.878	0.000110	0.0445
ENSMUSG00000058952	*Cfi*	3	Complement component factor i	1.10	0.000113	0.0446
ENSMUSG00000020427	*Igfbp3*	11	Insulin-like growth factor binding protein 3	0.854	0.000120	0.0453
ENSMUSG00000040653	*Ppp1r14 c*	10	Protein phosphatase 1, regulatory (inhibitor) subunit 14c	0.586	0.000123	0.0461
						
**E13.5**						
ENSMUSG00000041235	*Chd7*	4	Chromodomain helicase DNA binding protein 7	−1.58	1.41E−08	0.000489
ENSMUSG00000069171	*Nr2f1*	13	Nuclear receptor subfamily 2, group F, member	1–1.43	8.43E−06	0.0366
ENSMUSG00000023964	*Calcr*	6	Calcitonin receptor	−1.39	1.04E−05	0.0403
ENSMUSG00000073007	*Fam46d*	*X*	Family with sequence similarity 46, member D	−1.11	1.24E−05	0.0425
ENSMUSG00000033214	*Slitrk5*	14	SLIT and NTRK-like family, member	5–0.670	1.35E−05	0.0425
ENSMUSG00000028883	*Sema3 a*	5	Semaphorin 3A	−0.704	1.63E−05	0.0437
ENSMUSG00000025196	*Cpn1*	19	Carboxypeptidase N, polypeptide 1	1.93	2.32E−07	0.00404
ENSMUSG00000055312	*0610012H03Rik*	2	RIKEN cDNA 0610012H03 gene	0.911	3.92E−06	0.0273
ENSMUSG00000069792	*Wfdc17*	11	Predicted gene 11428	1.61	7.76E−06	0.0366

Chrom. indicates chromosome. Fold changes are given on a binary logarithmic scale (log_2_ FC), P.values of significance were calculated using a Bayes moderated *t* test, whilst adj.*p*.values of significance were calculated using the Benjamini and Hochberg False Discovery Rate process. Numerical values are shown to 3 significant figures.

## References

[bib1] Anderson R.H., Webb S., Brown N.A., Lamers W., Moorman A. (2003). Development of the heart: (2) septation of the atriums and ventricles. Heart.

[bib2] Buckingham M., Meilhac S., Zaffran S. (2005). Building the mammalian heart from two sources of myocardial cells. Nat. Rev. Genet..

[bib3] Bajpai R., Chen D.A., Rada-Iglesias A., Zhang J., Xiong Y., Helms J., Chang C.P., Zhao Y., Swigut T., Wysocka J. (2010). CHD7 cooperates with PBAF to control multipotent neural crest formation. Nature.

[bib4] Bosman E.A., Penn A.C., Ambrose J.C., Kettleborough R., Stemple D.L., Steel K.P. (2005). Multiple mutations in mouse Chd7 provide models for CHARGE syndrome. Hum. Mol. Genet..

[bib5] Bers D.M. (2002). Cardiac excitation–contraction coupling. Nature.

[bib6] Burney M.J., Johnston C., Wong K.Y., Teng S.W., Beglopoulos V., Stanton L.W., Williams B.P., Bithell A., Buckley N.J. (2013). An epigenetic signature of developmental potential in neural stem cells and early neurons. Stem Cells.

[bib7] Chang C.P., Bruneau B.G. (2012). Epigenetics and cardiovascular development. Annu. Rev. Physiol..

[bib8] Corsten-Janssen N., Kerstjens-Frederikse W.S., du Marchie Sarvaas G.J., Baardman M.E., Bakker M.K., Bergman J.E., Hove H.D., Heimdal K.R., Rustad C.F., Hennekam R.C. (2013). The cardiac phenotype in patients with a CHD7 mutation. Circ. Cardiovasc. Genet..

[bib9] Creazzo T.L., Godt R.E., Leatherbury L., Conway S.J., Kirby M.L. (1998). Role of cardiac neural crest cells in cardiovascular development. Annu. Rev. Physiol..

[bib10] Dobell A.R., Van Praagh R. (1996). The Holmes heart: historic associations and pathologic anatomy. Am. Heart J..

[bib11] Engelen E., Akinci U., Bryne J.C., Hou J., Gontan C., Moen M., Szumska D., Kockx C., van Ijcken W., Dekkers D.H. (2011). Sox2 cooperates with Chd7 to regulate genes that are mutated in human syndromes. Nat. Genet..

[bib12] Etchevers H.C., Amiel J., Lyonnet S., Saint-Jeannet J.P. (2006). Neural Crest Induction and Differentiation.

[bib13] Feiner L., Webber A.L., Brown C.B., Lu M.M., Jia L., Feinstein P., Mombaerts P., Epstein J.A., Raper J.A. (2001). Targeted disruption of semaphorin 3C leads to persistent truncus arteriosus and aortic arch interruption. Development.

[bib14] Feng Y., Valley M.T., Lazar J., Yang A.L., Bronson R.T., Firestein S., Coetzee W.A., Manley J.L. (2009). SRp38 regulates alternative splicing and is required for Ca(2+) handling in the embryonic heart. Dev. Cell.

[bib15] Gautier L., Cope L., Bolstad B.M., Irizarry R.A. (2004). affy—Analysis of Affymetrix GeneChip data at the probe level. Bioinformatics.

[bib16] Gentleman R.C., Carey V.J., Bates D.M., Bolstad B., Dettling M., Dudoit S., Ellis B., Gautier L., Ge Y., Gentry J. (2004). Bioconductor: open software development for computational biology and bioinformatics. Genome Biol..

[bib17] Hall B.D. (1979). Choanal atresia and associated multiple anomalies. J. Pediatr..

[bib18] Hurd E.A., Capers P.L., Blauwkamp M.N., Adams M.E., Raphael Y., Poucher H.K., Martin D.M. (2007). Loss of Chd7 function in gene-trapped reporter mice is embryonic lethal and associated with severe defects in multiple developing tissues. Mamm. Genome.

[bib19] Hasan W. (2013). Autonomic cardiac innervation: development and adult plasticity. Organogenesis.

[bib20] Huang D.W., Sherman B.T., Lempicki R.A. (2009). Bioinformatics enrichment tools: paths toward the comprehensive functional analysis of large gene lists. Nucleic Acids Res..

[bib21] Hanchate N.K., Giacobini P., Lhuillier P., Parkash J., Espy C., Fouveaut C., Leroy C., Baron S., Campagne C., Vanacker C. (2012). SEMA3A, a gene involved in axonal pathfinding, is mutated in patients with Kallmann syndrome. PLoS Genet..

[bib22] Hernandez-Miranda L.R., Cariboni A., Faux C., Ruhrberg C., Cho J.H., Cloutier J.F., Eickholt B.J., Parnavelas J.G., Andrews W.D. (2011). Robo1 regulates semaphorin signaling to guide the migration of cortical interneurons through the ventral forebrain. J. Neurosci..

[bib23] Ieda M., Kanazawa H., Kimura K., Hattori F., Ieda Y., Taniguchi M., Lee J.K., Matsumura K., Tomita Y., Miyoshi S. (2007). Sema3a maintains normal heart rhythm through sympathetic innervation patterning. Nat. Med..

[bib24] Kisanuki Y.Y., Hammer R.E., Miyazaki J., Williams S.C., Richardson J.A., Yanagisawa M. (2001). Tie2-Cre transgenic mice: a new model for endothelial cell-lineage analysis in vivo. Dev. Biol..

[bib25] Kramer S.G., Kidd T., Simpson J.H., Goodman C.S. (2001). Switching repulsion to attraction: changing responses to slit during transition in mesoderm migration. Science.

[bib26] Kubilus J.K., Linsenmayer T.F. (2010). Developmental guidance of embryonic corneal innervation: roles of Semaphorin3A and Slit2. Dev. Biol..

[bib27] Layman W.S., McEwen D.P., Beyer L.A., Lalani S.R., Fernbach S.D., Oh E., Swaroop A., Hegg C.C., Raphael Y., Martens J.R. (2009). Defects in neural stem cell proliferation and olfaction in Chd7 deficient mice indicate a mechanism for hyposmia in human CHARGE syndrome. Hum. Mol. Genet..

[bib28] Liu Y., Harmelink C., Peng Y., Chen Y., Wang Q., Jiao K. (2014). CHD7 interacts with BMP R-SMADs to epigenetically regulate cardiogenesis in mice. Hum. Mol. Genet..

[bib29] Merki E., Zamora M., Raya A., Kawakami Y., Wang J., Zhang X., Burch J., Kubalak S.W., Kaliman P., Izpisua Belmonte J.C. (2005). Epicardial retinoid X receptor alpha is required for myocardial growth and coronary artery formation. Proc. Natl. Acad. Sci. USA.

[bib30] Mommersteeg M.T., Yeh M.L., Parnavelas J.G., Andrews W.D. (2015). Disrupted Slit-Robo signalling results in membranous ventricular septum defects and bicuspid aortic valves. Cardiovasc. Res..

[bib31] Nam J., Onitsuka I., Hatch J., Uchida Y., Ray S., Huang S., Li W., Zang H., Ruiz-Lozano P., Mukouyama Y.S. (2013). Coronary veins determine the pattern of sympathetic innervation in the developing heart. Development.

[bib32] Puceat M., Jaconi M. (2005). Ca^2+^ signalling in cardiogenesis. Cell Calcium.

[bib33] Randall V., McCue K., Roberts C., Kyriakopoulou V., Beddow S., Barrett A.N., Vitelli F., Prescott K., Shaw-Smith C., Devriendt K. (2009). Great vessel development requires biallelic expression of Chd7 and Tbx1 in pharyngeal ectoderm in mice. J. Clin. Investig..

[bib34] Rothberg J.M., Hartley D.A., Walther Z., Artavanis-Tsakonas S. (1988). slit: An EGF-homologous locus of D. melanogaster involved in the development of the embryonic central nervous system. Cell.

[bib35] Schnetz M.P., Bartels C.F., Shastri K., Balasubramanian D., Zentner G.E., Balaji R., Zhang X., Song L., Wang Z., Laframboise T. (2009). Genomic distribution of CHD7 on chromatin tracks H3K4 methylation patterns. Genome Res..

[bib36] Schnetz M.P., Handoko L., Akhtar-Zaidi B., Bartels C.F., Pereira C.F., Fisher A.G., Adams D.J., Flicek P., Crawford G.E., Laframboise T. (2010). CHD7 targets active gene enhancer elements to modulate ES cell-specific gene expression. PLoS Genet..

[bib37] Saga Y., Miyagawa-Tomita S., Takagi A., Kitajima S., Miyazaki J., Inoue T. (1999). MesP1 is expressed in the heart precursor cells and required for the formation of a single heart tube. Development.

[bib38] Srinivasan R.S., Dillard M.E., Lagutin O.V., Lin F.J., Tsai S., Tsai M.J., Samokhvalov I.M., Oliver G. (2007). Lineage tracing demonstrates the venous origin of the mammalian lymphatic vasculature. Genes Dev..

[bib39] Schulz Y., Wehner P., Opitz L., Salinas-Riester G., Bongers E.M., van Ravenswaaij-Arts C.M., Wincent J., Schoumans J., Kohlhase J., Borchers A. (2014). CHD7, the gene mutated in CHARGE syndrome, regulates genes involved in neural crest cell guidance. Hum. Genet..

[bib40] Smyth G.K. (2004). Linear models and empirical bayes methods for assessing differential expression in microarray experiments. Stat. Appl. Genet. Mol. Biol..

[bib41] Tian Y., Morrisey E.E. (2012). Importance of myocyte–nonmyocyte interactions in cardiac development and disease. Circ. Res..

[bib42] Vissers L.E., van Ravenswaaij C.M., Admiraal R., Hurst J.A., de Vries B.B., Janssen I.M., van der Vliet W.A., Huys E.H., de Jong P.J., Hamel B.C. (2004). Mutations in a new member of the chromodomain gene family cause CHARGE syndrome. Nat. Genet..

[bib43] Van Nostrand J.L., Brady C.A., Jung H., Fuentes D.R., Kozak M.M., Johnson T.M., Lin C.Y., Lin C.J., Swiderski D.L., Vogel H. (2014). Inappropriate p53 activation during development induces features of CHARGE syndrome. Nature.

[bib44] Waldo K.L., Hutson M.R., Stadt H.A., Zdanowicz M., Zdanowicz J., Kirby M.L. (2005). Cardiac neural crest is necessary for normal addition of the myocardium to the arterial pole from the secondary heart field. Dev. Biol..

[bib45] Zentner G.E., Layman W.S., Martin D.M., Scacheri P.C. (2010). Molecular and phenotypic aspects of CHD7 mutation in CHARGE syndrome. Am. J. Med. Genet. A.

[bib46] Zaidi S., Choi M., Wakimoto H., Ma L., Jiang J., Overton J.D., Romano-Adesman A., Bjornson R.D., Breitbart R.E., Brown K.K. (2013). De novo mutations in histone-modifying genes in congenital heart disease. Nature.

[bib47] Zhang B., Dietrich U.M., Geng J.G., Bicknell R., Esko J.D., Wang L. (2009). Repulsive axon guidance molecule Slit3 is a novel angiogenic factor. Blood.

