# Supplementary material for: A critical role for the chromatin remodeller CHD7 in anterior mesoderm during cardiovascular development
Source: Dev Biol. 2015 Sep 1;405(1):82–95. doi: 10.1016/j.ydbio.2015.06.017 (PMC4534312; doi:10.1016/j.ydbio.2015.06.017)
Supplement: Supplementary file 1 — Supplementary material [file mmc1.pdf]

SUPPLEMENTAL MATERIAL – ***A Critical Role for the Chromatin Remodeller CHD7 in Anterior Mesoderm during Cardiovascular Development, Payne et. al.***

Supplemental Figures

Figure S1: Recombination of the R26R-LacZ reporter shows *Mesp1-Cre* activity is predominantly restricted to the anterior mesoderm

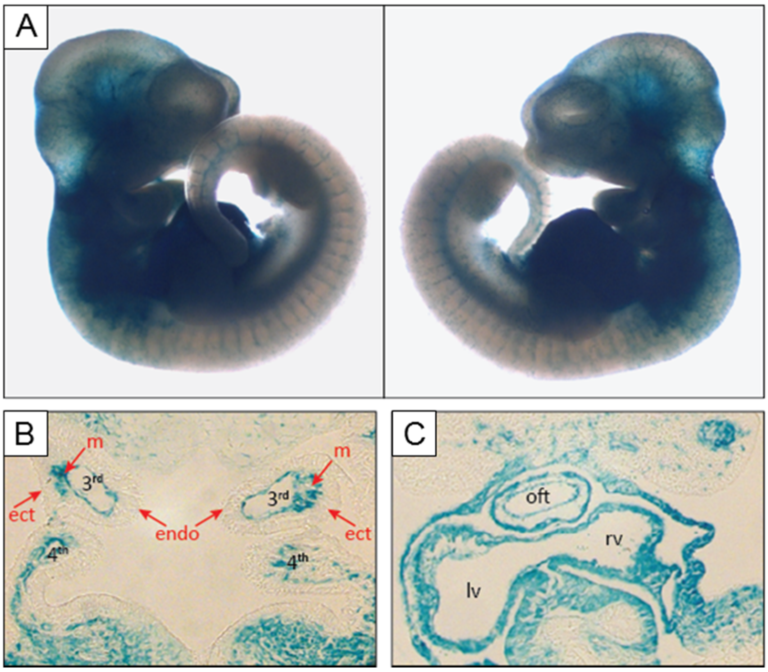

(A) Wholemount images of an E10.5 *R26R-LacZ;Mesp1-Cre* embryo after β-galactosidase staining, showing Cre activity in the cardiac, trunk and cranial mesoderm.

(B) Coronal sections through the 3rd and 4th pharyngeal arches showed β-galactosidase staining specifically in the mesoderm of the arches.

(C) Section through the heart confirmed *Mesp1-Cre* activity throughout the left and right ventricles and the outflow tract.

m indicates mesoderm; ect, ectoderm; endo, endoderm; oft, outflow tract; lv, left ventricle; rv, right ventricle.

Figure S2: Ablation of *Chd7* mRNA transcripts in *Chd7$^{fl/fl}$;Mesp1-Cre* hearts

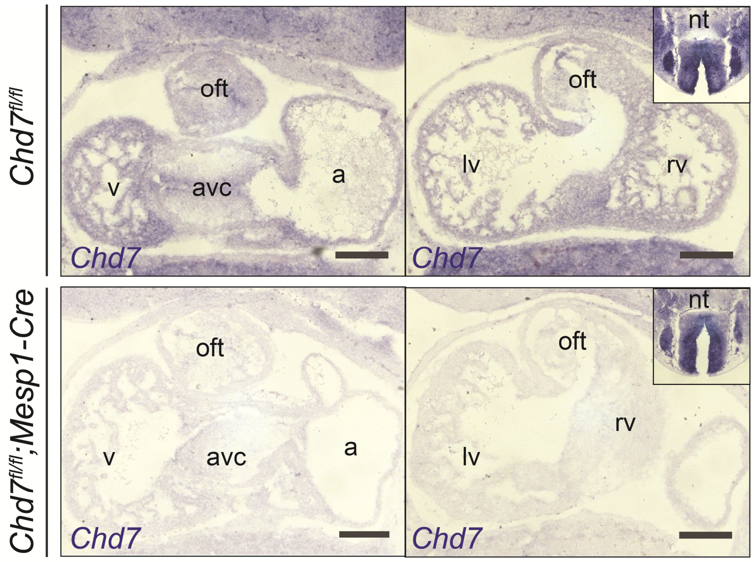

In situ hybridisation shows loss of *Chd7* mRNA on coronal sections through *Chd7$^{fl/fl}$;Mesp1-Cre* hearts (bottom panel) at E11.5, compared to *Chd7$^{fl/fl}$* littermate controls (top panel). Unchanged *Chd7* expression in the neural tube of the same embryos is also shown as an internal positive control (inserts).

Scale bars represent 0.2mm. a indicates atrium; v, ventricle; oft, outflow tract; avc, atrioventricular canal; nt, neural tube.

Figure S3: Addition of SHF progenitors to the OFT in *Chd7$^{fl/fl}$;Mesp1-Cre* hearts

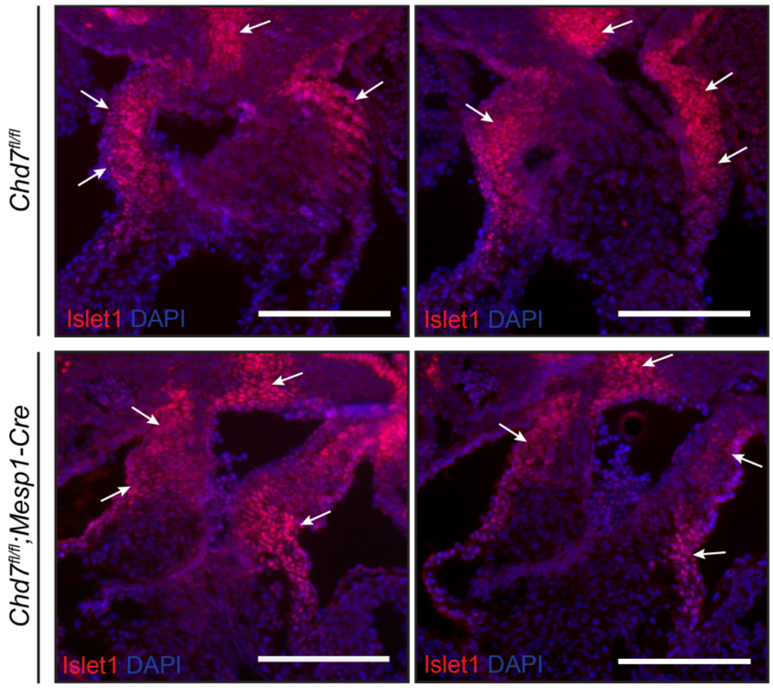

Immunofluorescence staining with anti-Islet1 antibody was performed on transverse sections through the OFT at E11.5, to visualise undifferentiated second heart field (SHF) progenitors being added to the arterial pole (highlighted with white arrows). Comparison of *Chd7$^{fl/fl}$* (top panel) and *Chd7$^{fl/fl}$;Mesp1-Cre* (bottom panel) sections showed very similar spatial distribution of Islet1-positive cells, indicating that localisation of these SHF progenitors to the developing OFT is not disrupted in *Chd7$^{fl/fl}$;Mesp1-Cre* hearts.

Scale bars represent 0.2mm.

Figure S4: Development of the epicardium is not disrupted in *Chd7$^{fl/fl}$;Mesp1-Cre* hearts

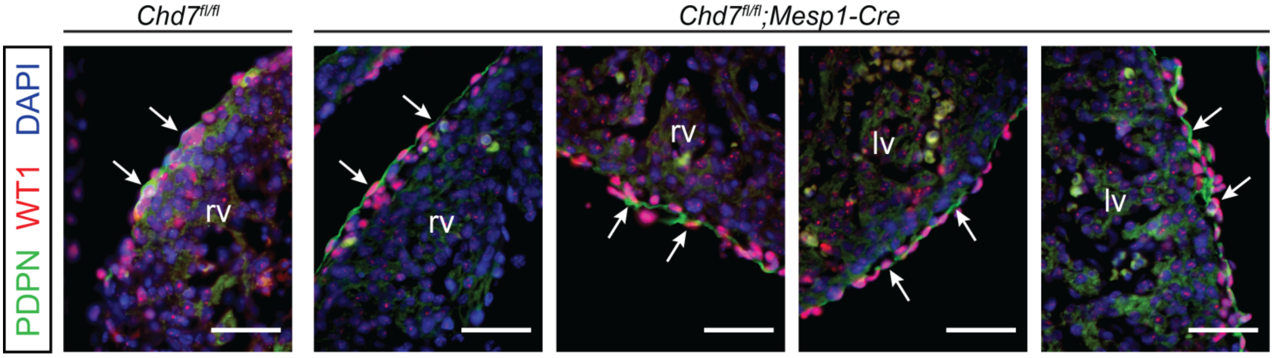

Immunofluorescence staining using antibodies for the epicardial markers Podoplanin (PDPN) and WT1 was performed on transverse heart sections at E13.5. The panel on the left shows normal expression of these markers in the epicardium of a *Chd7$^{fl/fl}$* control (arrows), with similar expression and morphology seen throughout the *Chd7$^{fl/fl}$;Mesp1-Cre* heart.

Scale bars represent 0.05mm. Rv indicates right ventricle; lv, left ventricle.

Figure S5: No hypocellular defect is seen at E10.5 in *Chd7*$^{fl/fl}$*;Mesp1-Cre* hearts

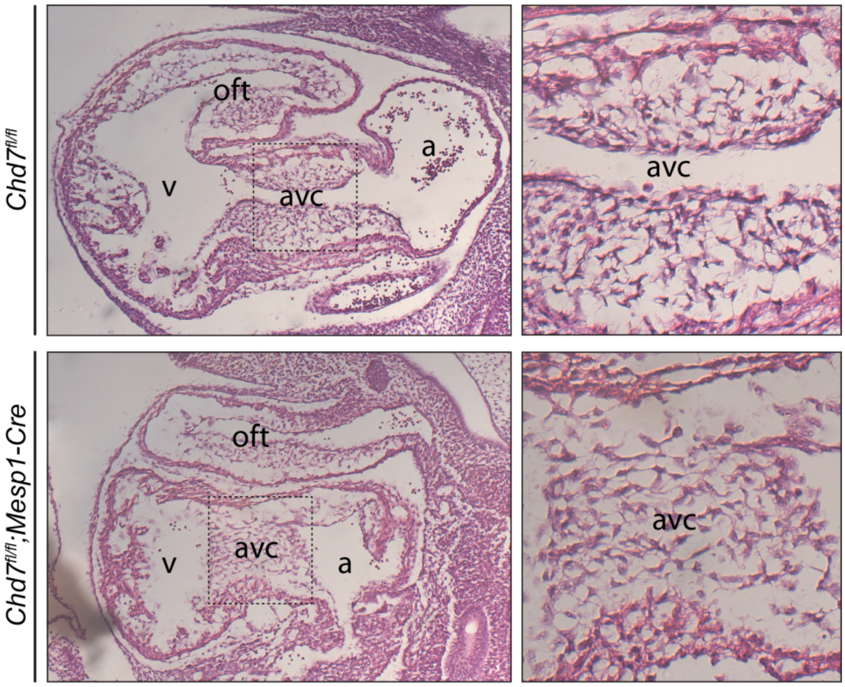

H&E coronal sections through a control *Chd7*$^{fl/fl}$ and conditional mutant *Chd7*$^{fl/fl}$*;Mesp1-Cre* heart at E10.5. As seen in E11.5 hearts, whilst overall alignment defects can be seen, there is no hypocellularity defect in the cushions or gross tissue loss.

Oft indicates outflow tract; v, ventricle; a, atrium; avc, atrioventricular canal.

Figure S6: CHD7 protein levels in the developing heart

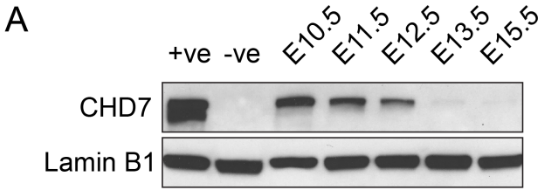

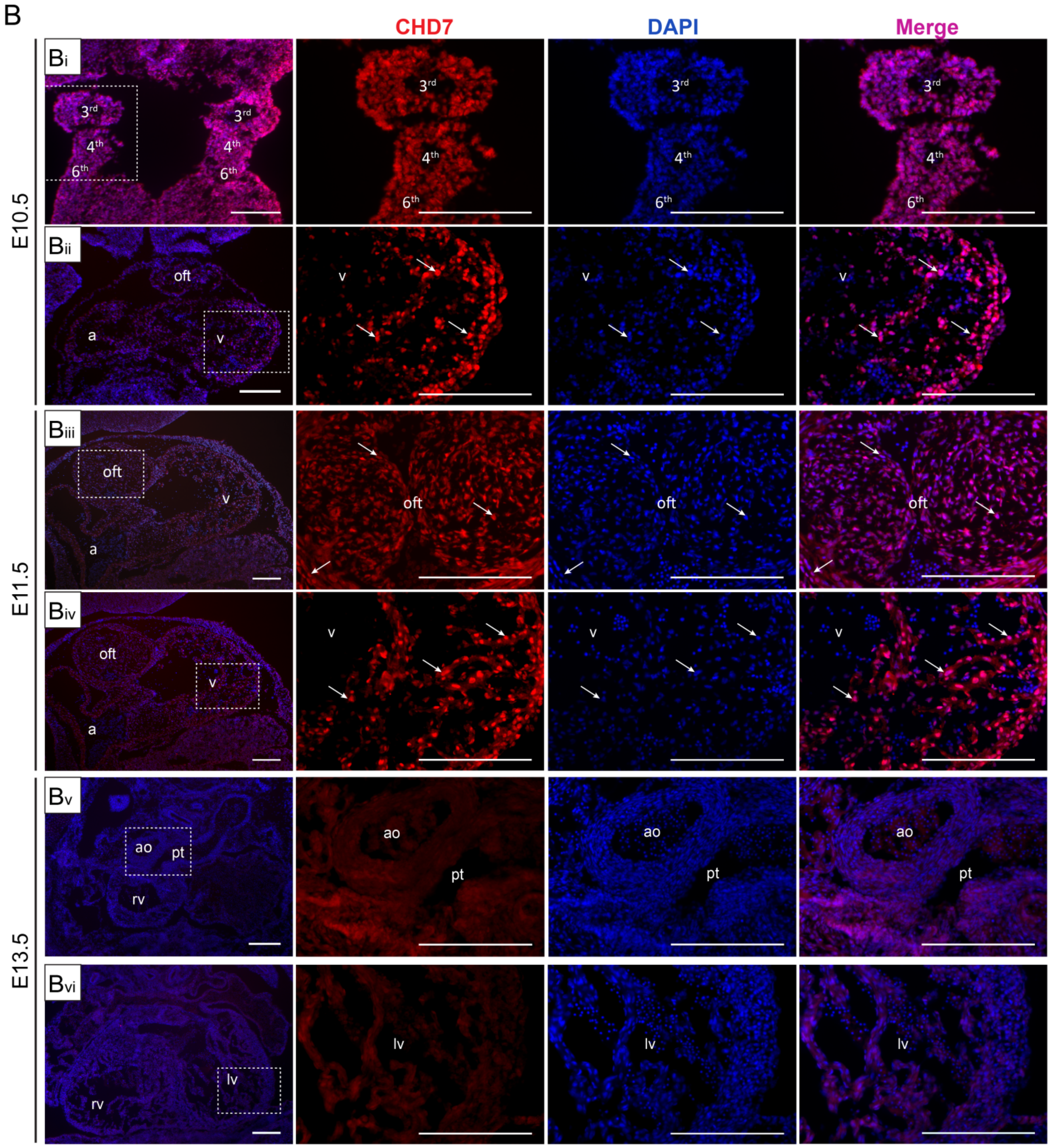

<u>SUPPLEMENTAL MATERIAL</u> – ***A Critical Role for the Chromatin Remodeller CHD7 in Anterior Mesoderm during Cardiovascular Development, Payne et. al.***

Figure S6: CHD7 protein levels in the developing heart

(A) Western blot time course showing CHD7 levels in whole heart protein extracts between E10.5 to E15.5. The high level of CHD7 protein at E10.5 then then decreases until it is barely detectable by E13.5. The +ve lane is protein extracted from murine ESCs and the –ve lane is protein from HEK293T cells. The Western blot for the nuclear protein Lamin B1 is also shown as a loading control.

(B) Immunofluorescence on cryosections through wild-type CD1 hearts between developmental stages E10.5 to E13.3. Ubiquitous CHD7 expression was seen at E10.5 in the pharyngeal region (Bi) and throughout the heart (Bii). Higher magnification imaging showed overlap with DAPI staining, confirming its nuclear localisation. Similar widespread CHD7 expression was seen at E11.5 in the endocardial cushions and endothelial lining of the outflow tract (Biii) and again in the myocardium and endocardium of the ventricles (Biv). However, at E13.5, CHD7 protein could no longer be detected anywhere in the OFT region (Bv) or the heart (Bvi) by immunohistochemistry.

All scale bars represent 0.2mm. 3rd, 4th and 6th label the pharyngeal arch arteries; oft, outflow tract; a, atrium; rv, right ventricle; lv, left ventricle; ao, aorta; pt, pulmonary trunk.

__SUPPLEMENTAL MATERIAL__ – ***A Critical Role for the Chromatin Remodeller CHD7 in Anterior Mesoderm during Cardiovascular Development, Payne et. al.***

Supplemental Tables

Table S1: Embryonic lethality of the *Chd7$^{fl/fl}$;Mesp1-Cre* phenotype

Developmental Stage:	Genotype:	Observed:	Expected:	Necrotic:
**E10.5**	*Chd7$^{fl/+}$*	22	18	0
	Chd7$^{fl/fl}$	19	18	0
	Chd7$^{fl/+}$;Mesp1-Cre	15	18	0
	Chd7$^{fl/fl}$;Mesp1-Cre	**15**	**18**	**0**
**E13.5**	*Chd7$^{fl/+}$*	17	14	0
	Chd7$^{fl/fl}$	18	14	0
	Chd7$^{fl/+}$;Mesp1-Cre	8	14	0
	Chd7$^{fl/fl}$;Mesp1-Cre	**13**	**14**	**0**
**E15.5**	*Chd7$^{fl/+}$*	36	36	0
	Chd7$^{fl/fl}$	43	36	1
	Chd7$^{fl/+}$;Mesp1-Cre	36	36	1
	Chd7$^{fl/fl}$;Mesp1-Cre	**27**	**36**	**7****
**E18.5**	*Chd7$^{fl/+}$*	13	10	0
	Chd7$^{fl/fl}$	13	10	0
	Chd7$^{fl/+}$;Mesp1-Cre	13	10	0
	Chd7$^{fl/fl}$;Mesp1-Cre	**2***	**10**	**1**
**P10**	*Chd7$^{fl/+}$*	12	9	-
	Chd7$^{fl/fl}$	8	9	-
	Chd7$^{fl/+}$;Mesp1-Cre	15	9	-
	Chd7$^{fl/fl}$;Mesp1-Cre	**0****	**9**	**-**

Expected numbers are based on Mendelian ratios, rounded to the nearest whole number.

** p <0.05, ** p<0.01, based on Chi squared analysis.*

<u>SUPPLEMENTAL MATERIAL</u> – ***A Critical Role for the Chromatin Remodeller CHD7 in Anterior Mesoderm during Cardiovascular Development, Payne et. al.***

Table S2: Full lists of genes with altered expression ($log_2FC > 0.5$, $p < 0.05$) in the heart at E11.5 or E13.5 following mesodermal deletion of *Chd7*

Please see the attached Excel spreadsheet. Different tabs contain the lists of genes identified as being downregulated or upregulated by microarray analysis of dissected hearts at E11.5 and E13.5. Within each table, those genes with an adjusted p value < 0.05 following multiple testing correction (using the Benjamini-Hochberg procedure) are shaded pale blue.

Table S3: GO Term Clusters for Genes Downregulated at E11.5 in *Chd7$^{fl/fl}$;Mesp1-Cre* Hearts

GO Term:	Count:	P Value:	Genes:
Neuron projection development	5	0.022988	*ROBO2, SEMA3A, LMX1A, GRIN3A, SLIT2*
Leukocyte differentiation	4	0.033534	*CALCR, IKZF1, MITF, IL15*
Hemopoiesis	5	0.035969	*CALCR, IKZF1, MITF, IL15, MB*
Regulation of neurotransmitter levels	3	0.037981	*ACHE, SNAP25, LIN7A*
Eye development	4	0.043124	*ACHE, CHD7, IKZF1, MITF*
Axonogenesis	4	0.047306	*ROBO2, SEMA3A, LMX1A, SLIT2*
Forebrain development	4	0.050202	*DKK1, IKZF1, LMX1A, NR2F1*
Hemopoietic or lymphoid organ development	5	0.050824	*CALCR, IKZF1, MITF, IL15, MB*
Negative regulation of signal transduction	4	0.053183	*DKK1, RGS5, RGS18, FRZB*
Brain development	5	0.054712	*DKK1, IKZF1, ROBO2, LMX1A, NR2F1*
Neuron projection morphogenesis	4	0.057027	*ROBO2, SEMA3A, LMX1A, SLIT2*
Myeloid cell differentiation	3	0.082361	*CALCR, MITF, MB*
Cell morphogenesis involved in differentiation	4	0.088384	*ROBO2, SEMA3A, LMX1A, SLIT2*
Lymph node development	2	0.090398	*IKZF1, IL15*
Regulation of neuron differentiation	3	0.091322	*ROBO2, SEMA3A, LMX1A*

SUPPLEMENTAL MATERIAL – ***A Critical Role for the Chromatin Remodeller CHD7 in Anterior Mesoderm during Cardiovascular Development, Payne et. al.***

Table S4: GO Term Clusters for Genes Upregulated at E11.5 in *Chd7$^{fl/fl}$;Mesp1-Cre* Hearts

GO Term:	Count:	P Value:	Genes:
Tissue development	10	0.00112	*SHOX2, ALDH1A2, ADM, RXFP1, HOXA5, IRF6, EDN1, SPRR2B, MET, NR4A3*
G-protein coupled receptor signalling pathway	16	0.007626	*RXFP1, PTH2R, OLFR538, OLFR1413, EDN1, OLFR559, OLFR735, OLFR1309, SSTR4, OLFR1042, OLFR1156, RGS6, OLFR878, OLFR1126, OLFR429, OLFR366*
Cell surface receptor linked signal transduction	19	0.009247	*PTH2R, RXFP1, OLFR538, OLFR1413, MET, EDN1, OLFR559, OLFR735, OLFR1309, SSTR4, SFRP5, OLFR1042, OLFR1156, RGS6, OLFR1126, OLFR878, OLFR429, GFRA2, OLFR366*
Mesenchyme development	3	0.018267	*ALDH1A2, HOXA5, EDN1*
Epithelium development	5	0.025821	*ALDH1A2, ADM, HOXA5, IRF6, SPRR2B*
Sensory perception	12	0.026766	*OLFR1042, OLFR1156, TRPM8, OLFR538, OLFR1413, OLFR559, OLFR878, OLFR1126, OLFR429, OLFR1309, OLFR735, OLFR366*
Embryonic organ morphogenesis	4	0.029166	*SHOX2, HOXA5, EDN1, NR4A3*
Cartilage development	3	0.041613	*SHOX2, HOXA5, EDN1*
Gland development	4	0.048366	*RXFP1, HOXA5, IRF6, MET*
Myotube differentiation	2	0.048767	*MET, MYH9*
Mammary gland development	3	0.049603	*RXFP1, IRF6, MET*
Negative regulation of cell proliferation	4	0.065965	*IRF6, HMOX1, IGFBP3, ALOX8*
Tissue morphogenesis	4	0.076102	*ALDH1A2, ADM, HOXA5, NR4A3*
Lung development	3	0.077653	*ALDH1A2, RXFP1, HOXA5*
Blood vessel development	4	0.080648	*ALDH1A2, HMOX1, EDN1, MYH9*

SUPPLEMENTAL MATERIAL – ***A Critical Role for the Chromatin Remodeller CHD7 in Anterior Mesoderm during Cardiovascular Development, Payne et. al.***

Table S5: GO term Clusters for Genes Downregulated at E13.5 in *Chd7$^{fl/fl}$;Mesp1-Cre* Hearts

GO Term:	Count:	P Value:	Genes:
Blood coagulation	6	3.07E-05	*F2RL2, GP5, PLEK, P2RX1, TREML1, GP9*
Platelet activation	3	0.003065	*PLEK, P2RX1, TREML1*
Cell surface receptor linked signal transduction	23	0.006099	*CALCR, F2RL2, GABRG3, ADAMTS19, GABRA1, PLEK, GABRA4, FST, BEX1, MSTN, RGS18, ITGB3, GPRC6A, OLFR173, GPR22, FZD10, CHRM2, P2RY1, MTNR1B, CNTN1, ANGPT1, ITGA2B, CLEC1B*
Axonogenesis	5	0.010275	*SLITRK3, ETV1, SEMA3A, NRN1, SLITRK5*
Platelet degranulation	2	0.010403	*PLEK, P2RX1*
Cell morphogenesis in neuron differentiation	5	0.014903	*SLITRK3, ETV1, SEMA3A, NRN1, SLITRK5*
Regulation of receptor recycling	2	0.025809	*ACHE, GRIA2*
Regulation of hair follicle development	2	0.035948	*INHBA, FST*
Blood vessel development	5	0.038304	*CHD7, MEOX2, PRRX1, SEMA3C, ANGPT1*
Generation of neurons	7	0.044383	*SLITRK3, BEX1, ETV1, SEMA3A, NRN1, SLITRK5, NR2F1*
Integrin-mediated signalling pathway	3	0.059648	*ADAMTS19, ITGB3, ITGA2B*
Inorganic anion transport	3	0.069607	*GABRG3, GABRA1, GABRA4*
Blood vessel morphogenesis	4	0.084404	*MEOX2, PRRX1, SEMA3C, ANGPT1*
Branching in salivary gland morphogenesis	2	0.089884	*SEMA3C, SEMA3A*
Positive regulation of ion transport	2	0.099366	*P2RX1, P2RY1*
Blood coagulation	6	3.07E-05	*F2RL2, GP5, PLEK, P2RX1, TREML1, GP9*

Table S6: GO Term Clusters for Genes Upregulated at E13.5 in *Chd7$^{fl/fl}$;Mesp1-Cre* Hearts

GO Term:	Count:	P Value:	Genes:
Unsaturated fatty acid metabolic process	3	0.008325	*EPHX2, ALOX8, MGST2*

<u>SUPPLEMENTAL MATERIAL</u> – ***A Critical Role for the Chromatin Remodeller CHD7 in Anterior Mesoderm during Cardiovascular Development, Payne et. al.***

Table S7: Microarray results for Class 3 Semaphorin, Slit-Robo and Calcium Handling Genes ($log_2FC < 0.5$, $p < 0.05$)

Symbol:	Gene name:	E11.5 log_2FC:	E11.5 P Value:	E13.5 log_2FC:	E13.5 P Value:
Sema3A	semaphorin 3A	-0.89409	9.87E-07	-0.70435	1.63E-05
Sema3C	semaphorin 3C	-0.63997	0.001107	-0.65304	0.000936
Sema3E	semaphorin 3E	NS	NS	0.554481	0.030159
Robo2	roundabout homolog 2 (Drosophila)	-0.90673	2.38E-05	NS	NS
Slit2	slit homolog 2 (Drosophila)	-0.98707	2.44E-08	-0.53024	4.66E-05
Slitrk3	SLIT and NTRK-like family, member 3	NS	NS	-0.65121	0.018027
Slitrk4	SLIT and NTRK-like family, member 4	NS	NS	-0.54174	0.01459
Slitrk5	SLIT and NTRK-like family, member 5	NS	NS	-0.67045	1.35E-05
Cacna1e	calcium channel, voltage-dependent, R type, alpha 1E subunit	-0.504	0.000393	NS	NS
Cacna2d3	calcium channel, voltage-dependent, alpha2/delta subunit 3	-0.69777	0.004466	NS	NS
Cacng7	calcium channel, voltage-dependent, gamma subunit 7	-0.60886	0.0016	NS	NS
Casq2	calsequestrin 2	1.691003	1.98E-08	0.556697	0.003187
Trdn	triadin	-1.0781	6.84E-07	-0.8	2.28E-05
Ryr3	ryanodine receptor 3	NS	NS	-0.52855	0.000215

NS indicates not significant

SUPPLEMENTAL MATERIAL – ***A Critical Role for the Chromatin Remodeller CHD7 in Anterior Mesoderm during Cardiovascular Development, Payne et. al.***

Supplemental Methods:

Histology

Dissected embryos were fixed overnight in 4% PFA/PBS and then dehydrated through an ethanol series, cleared by washing in histoclear (National Diagnostics), and embedded in paraffin after overnight washes. A microtome was used to section embryos to 12-15µm thickness in a coronal or transverse plane, and sections were transferred onto 3-triethoxysilylpropylamine (TESPA)-coated glass slides. For morphological examination, sections were rehydrated and stained using freshly-filtered Mayer's Hemotoxylin solution (Sigma-Aldrich) followed by aqueous Eosin solution (Sigma-Aldrich). Slides were mounted with glass coverslips using DPX solution (Merck).

For quantitation of the thickness and compaction of the ventricular walls in E15.5 hearts, three different non-consecutive transverse sections through 5 control and 5 mutant hearts were examined, with ImageJ used to take measurements of the width (in mm) across the compact layer and trabeculae at three different points around both the left and right ventricles in each section. The mean widths were then calculated and plotted with the standard error, and the mean percentage of the total ventricular wall thickness that each layer comprised was calculated. Statistical analysis was performed using the unpaired student t test.

Intracardiac Ink Injection

After overnight fixation of embryos in 4% PFA/PBS at 4°C, E10.5 outflow tracts were injected with India ink (Pélican) using a pulled glass capillary to fill the 3rd, 4th and 6th pharyngeal arch arteries (as previously described [1]). Fixed E15.5 embryos were opened to view the chest cavity and India ink injected into the cardiac ventricles when insufficient blood remained in the great vessels to view their structure.

Immunofluorescence on Wax Sections

Tissue sections were prepared in the same way as for Hemotoxylin and Eosin staining (see above). Slides were de-waxed by pre-warming at 60°C followed by two 10 minute washes in histoclear, and rehydrated by 5 minute washes through an EtOH series down to 30% EtOH. Antigen retrieval was carried out by incubation in pre-warmed Target Retrieval Solution (Dako) for 30 minutes at 94-96°C, 20 minutes at room temperature, and then 5 minutes under running water. Sections were then incubated for 1 hour at room temperature in blocking solution (1% BSA, 2% FBS (Life Technologies), PBS) followed by incubation overnight at 4°C in primary antibodies. Slides were then rinsed in PBS and incubated in secondary antibodies (Invitrogen) diluted 1:500 in blocking solution for 1 hour at room temperature, and then, where required, tertiary antibody diluted 1:500 for another 30 minutes. Finally, slides were rinsed further in PBS, stained with DAPI and mounted with glass coverslips using VECTASHIELD® mounting medium.

Primary antibodies used were mouse monoclonal to Islet1 (40.2D6, Developmental Studies Hybridoma Bank) diluted 1:25, golden Syrian hamster monoclonal to Podoplanin (NB600-1015, Novus) diluted 1:100, and rabbit monoclonal to WT1 (ab89901, Abcam) diluted 1:100 in blocking solution. Secondary antibodies were Biotinylated goat anti-mouse IgG (Vector Laboratories), AlexaFluor® 488 goat anti-Syrian hamster IgG (Life Technologies), and AlexaFluor® 594 goat anti-rabbit IgG (Life Technologies). The tertiary antibody was AlexaFluor® 594-conjugated Streptavadin.

Immunofluorescence on Cryosections

Embryos were fixed in 4% PFA/PBS at 4°C for 30-60 minutes, washed in PBS and then incubated overnight in 30% sucrose/PBS at 4°C. This was followed by incubation in a 50/50 mixture of 30% sucrose/OCT embedding matrix (Fisher) until the embryos sank, and then incubation in 100% OCT. Samples were mounted into moulds on dry ice and stored at -80°C before crysectioning at 10µM thickness onto Superfrost Plus slides (VWR).

For immunofluorescence staining, sections were brought to room temperature, rinsed twice in PBS and permeabilised with 0.5% Triton X-100 (Sigma-Aldrich)/PBS for 10 minutes before further rinsing in PBS. Sections were incubated for 1 hour in blocking solution (1% BSA, 10% goat serum, 0.1% Triton X-100) in a humidified chamber, then incubated overnight at 4°C in rabbit polyclonal to CHD7 (NBP1-77393, Novus Biologicals) diluted 1:200 in blocking solution. Following washes in 0.1% Triton X-100/PBS and then PBS, slides were incubated in AlexaFluor® 594 goat anti-rabbit-IgG (Life Technologies) diluted 1:1000 in blocking solution before finally washing again and counter-staining with DAPI.

Visualising Parasympathetic Innervation on Wholemount Hearts

E15.0 hearts were dissected into PBS before fixation for 20-30 minutes in 4% PFA/PBS at room temperature. Hearts were then permeabilised by incubation in 0.5% Triton-X/PBS for 5 minutes, washed in PBS and stored overnight at 4°C in blocking solution (1% BSA, 10% sheep serum, 0.1% Triton-X). Hearts of the required genotype were then incubated overnight at 4°C with rotation in rabbit monoclonal antibody to Neurofilament-66 (04-1032, Millipore) diluted 1:50 in blocking solution, washed in PBS at room temperature, incubated overnight at 4°C in AlexaFluor® 594 goat anti-rabbit-IgG (Life Technologies) diluted 1:1000 in blocking solution, and finally washed again in PBS. The dorsal views of the hearts were photographed using a Zeiss SteREO Lumar.V12 microscope equipped with an AxioCam HRc camera and UV lamp and images processed using ImageJ.

Visualising Coronary Veins on Wholemount Hearts

E15.5 hearts were dissected into PBS, fixed in 4% PFA/PBS for 40 minutes at room temperature, washed in PBS and then dehydrated through a methanol (MeOH) series and stored at -20°C in 100% MeOH until genotyped. After rehydration to PBS, hearts were permeabilised by washing twice in 0.1% Tween-20/PBS (PBT) before incubation for two hours at room temperature in blocking solution (10% goat serum in PBT) and then overnight incubation in rat monoclonal to Endomucin (sc-65495, Santa Cruz) diluted 1:50 in blocking solution. Samples were washed in PBT at room temperature and then incubated overnight at 4°C in secondary Alexafluor-594-conjugated anti-rat-IgG antibody (Invitrogen) diluted 1:1000 in blocking solution. Hearts were again photographed using a Zeiss SteREO Lumar.V12 microscope equipped with an AxioCam HRc camera and UV lamp and images processed using ImageJ.

qRT-PCR

Total RNA was prepared using the same method as for the microarrays. The High-Capacity Reverse Transcription Kit with RNase Inhibitor (Applied Biosystems) was used to make cDNA, of which 600ng per lane was loaded on custom-designed TaqMan® Array Micro Fluidic Cards, according to manufacturer's instructions. Cards were run on an Applied Biosystems 7900HT Fast Real-Time PCR System using a

TaqMan® Array Micro Fluidic Card Thermal Cycling Block with SDS Software v2.1. The comparative C_T ($\Delta\Delta C_T$) method was used for determination of gene expression changes, and p-values calculated using unpaired Student's t-Test.

RNA Probe Preparation

Plasmids were linearised overnight using the appropriate restriction enzymes for generating antisense probes, and extracted from a 1% agarose gel using the QIAquick Gel Extraction Kit (Qiagen). 1µg of linearised plasmid was used for *in vitro* transcription of probes using a DIG RNA labelling kit (Roche). Probes were precipitated by addition of 2µl 0.5M EDTA (pH 8), 5µl 4M LiCl and 150µl ethanol to the reaction and extracted by centrifugation.

In Situ Hybridisation on Paraffin Sections

Paraffin sections were prepared as previously described. Slides were rehydrated then incubated in 20µg/ml Proteinase K (Sigma-Aldrich) for 8 minutes, washed in 2mg/ml glycine then PBS, and fixed in 4% PFA/PBS for 20 minutes. Following further PBS washes they were incubated for 1 hour at 70°C in a humidified chamber in hybridisation buffer (50% formamide, 5xSSC pH 4.5, 50µg/ml yeast RNA, 1% SDS, 50µg/ml heparin) followed by overnight incubation in hybridisation buffer containing 1µg/ml antisense RNA probe. Slides were then rinsed twice in 2x SSC buffer pH4.5, followed by three washes at 65°C in Solution I (50% formamide, 5x SSC pH4.5, 1% SDS), two washes in Solution II (50% formamide, 2x SSC pH4.5) and finally two washes at room temperature in MABT (0.1M maleic acid, 0.15M NaCl, 0.01% Tween-20, 2mM Levamisole (Sigma-Aldrich), pH7.5). Slides were then incubated in blocking solution (2% Boehringer Blocking Reagent (Roche), 10% sheep serum in MABT) for 1 hour followed by overnight incubation at 4°C with an alkaline-phosphatase (AP) conjugated anti-DIG antibody (Roche) diluted 1:2000 in blocking buffer. Following further washes in MABT and AP buffer (100mM Tris, pH 9.5, 50mM $MgCl_2$, 100mM NaCl, 0.1% Tween-20, 2mM Levamisol), AP activity was detected using BM Purple (Roche).

SUPPLEMENTAL MATERIAL – ***A Critical Role for the Chromatin Remodeller CHD7 in Anterior Mesoderm during Cardiovascular Development, Payne et. al.***

Primers used in ChIP and DNase I assays

**Primer:**	**Sequence Forward:**	**Sequence Reverse:**	**Annealing temp (°C):**
Sema3c-1	AGCCGGACTCAGATTTTCAG	GCGTGGGCTCCTAAGAAAAT	60
Sema3c-2	TCCAGACTAGACCTGCTTGTCA	AAAGGGAGGCCAGGTACTGT	60
Sema3c-3	CAGCATCATTTTTATGGGATGA	CAGGCAGCAAGCAGTAGGA	60
Sema3c-4	TCATGTCAGGTCTGCTCCAA	ATCAGCCCAAACAAAATGCT	60
Sema3c-5	GAGAGGGTCTTGCCTCTCCT	TGCACAGCCATTAACAGACC	60
Sema3c-6	GCTCAGTGCTTCCTGCTTCT	CACAGTTGATCCCTCATTGG	60
**Negative region**	AGGAGGGAAGGAGGAACAAA	GTGGCATCAACACAACGAAC	60
***Gapdh* promoter**	CTGGCACTGCACAAGAAGAT	GGTCCAAAGAGAGGGAGGAG	66
***Nanog* promoter**	GGCTGATTTGGTTGGTGTCT	TTCCCAGAATTCGATGCTTC	64
***Sema3c* promoter**	TCGGATATTTACAGCGTAGCC	GCCTTTGGCAGTGAACAGTC	60

The Sema3c primers 1-6 and the negative region primer were used for the ChIP-PCR (Figure 6D,E), whilst the Gapdh, Nanog and Sema3c promoter primers were used in the RT-PCR for the DNase I assay (Figure 6F).

Supplemental References

1. Lindsay EA, Botta A, Jurecic V, Carattini-Rivera S, Cheah YC, Rosenblatt HM, Bradley A, Baldini A (1999) Congenital heart disease in mice deficient for the DiGeorge syndrome region. *Nature* **401:** 379-383
